# Substrate identification of putative NCS1 and NCS2 nucleobase transporters in *Pseudomonas aeruginosa*

**DOI:** 10.1128/mbio.02434-24

**Published:** 2024-10-30

**Authors:** Corey Kennelly, Arthur Prindle

**Affiliations:** 1 Department of Biochemistry and Molecular Genetics, Feinberg School of Medicine, Northwestern University, Chicago, Illinois, USA; 2 Center for Synthetic Biology, Northwestern University, Chicago, Illinois, USA; 3 Department of Chemical and Biological Engineering, Northwestern University, Evanston, Illinois, USA; 4 Chan Zuckerberg Biohub Chicago, Chicago, Illinois, USA; Florida International University, Miami, Florida, USA; University of Washington, Seattle, Washington, USA

**Keywords:** *Pseudomonas aeruginosa*, nucleobase, purines, pyrimidines, transporters

## Abstract

*Pseudomonas aeruginosa* is an opportunistic pathogen that can salvage nucleobases from the environment to conserve nutrients that would otherwise be spent on *de novo* nucleotide biosynthesis. However, little is known regarding the substrate specificity of the 13 putative nucleobase transporters in *P. aeruginosa*. Here, using a combination of genetic and chemical approaches, we report substrate identifications for 10 putative nucleobase transporters in *P. aeruginosa*. Specifically, we individually expressed each transporter in a genetic background lacking all 13 putative nucleobase transporters and quantified growth on a panel of 10 nucleobases as sole nitrogen sources. We confirmed these expression-based substrate identifications using targeted genetic knockouts. In a complementary approach, we utilized four toxic nucleobase antimetabolites to characterize antimicrobial activity in these same strains. We identified the sole allantoin transporter as well as transporters for guanine, xanthine, uric acid, cytosine, thymine, uracil, and dihydrouracil. Furthermore, we associated at least five nucleobase transporters with hypoxanthine, which has been recently reported to be an antibiofilm cue in *P. aeruginosa*. These results provide an initial characterization of the putative nucleobase transporters in *P. aeruginosa*, significantly advancing our understanding of nucleobase transport in this clinically relevant organism.

**IMPORTANCE:**

*Pseudomonas aeruginosa* is a frequently multidrug-resistant opportunistic pathogen and one of the most common causes of healthcare-acquired infections. While nucleobases are known to support growth in nutrient-limited conditions, recent work showed that adenine and hypoxanthine can also decrease *P. aeruginosa* biofilm formation by disrupting c-di-GMP metabolism. Thus, nucleobase transport may be relevant to multiple aspects of *P. aeruginosa* biology and pathogenesis. However, there is currently little known about the transport of nucleobases in *P. aeruginosa*. Our work reports initial substrate identifications for 10 putative nucleobase transporters in *P. aeruginosa*, providing new tools to address previously difficult-to-test hypotheses relating to nucleobase transport in this organism.

## INTRODUCTION

Members of the nucleobase-cation symporter-1 (NCS1) and nucleobase-cation symporter-2 (NCS2) transporter families are widely present in bacteria, archaea, and eukarya (1, 2). While thousands of these transporters have been identified through sequencing, relatively few have been characterized experimentally, and only a fraction of these characterized transporters are of bacterial origin (1, 2). Most knowledge regarding bacterial NCS1 and NCS2 transporters has been gained from studying *Escherichia coli* and *Bacillus subtilis* (3–15), although their transporters have not all been experimentally characterized. Many transporters from other bacteria do not share high similarity with these characterized transporters, and even transporters similar by nucleotide or amino acid sequence can exhibit different substrate profiles (16–23). Thus, substrate identification of putative NCS1 and NCS2 transporters remains an important goal in many bacterial species.

NCS1 and NCS2 transporters generally catalyze the uptake of purine and pyrimidine nucleobases and structurally similar molecules (1, 2). Once these molecules are internalized, cells can use salvage pathways to convert them into late intermediates of nucleotide biosynthesis (24, 25). This process saves energy that would otherwise be consumed by *de novo* nucleotide biosynthesis (25–27). Cells can also use degradation pathways to break these molecules down for their carbon and nitrogen contents (28). While nucleobases are not generally thought to be the preferred source of carbon or nitrogen for most organisms, many organisms can nevertheless utilize them as carbon and/or nitrogen sources when necessary (28). Thus, nucleobase transport may be beneficial to bacterial survival in situations where nutrients are limited.

The rise in bacterial resistance to antimicrobial treatment has generated interest in the potential use of nucleobase analogs for their antimicrobial and/or antivirulence properties (29–33). Nucleobase transporters are likely points of entry for purine and pyrimidine analogs and are therefore prime candidates for inactivation in the development of resistance to these compounds (34). Since antimetabolites share structural similarities with metabolites, inferences regarding potential native substrates for transporters can additionally be made once antimetabolite substrate profiles have been determined. Thus, substrate identification of nucleobase transporters in clinically relevant organisms may facilitate the development of new antimicrobials and understanding of their likely resistance mechanisms.

With this context, *Pseudomonas aeruginosa* is a frequently multidrug-resistant gram-negative opportunistic pathogen known for its ability to survive in multiple environments and metabolize a diverse set of substrates (35–37). Accordingly, *P. aeruginosa* encodes six genes annotated as putative NCS1 transporters and seven genes annotated as putative NCS2 transporters (38). In this work, we leveraged the ability of *P. aeruginosa* to utilize nucleobases as sole nitrogen sources along with a comprehensive set of knockout strains and expression vectors to perform growth-based experiments to characterize substrate specificity of these 13 transporters. In parallel, we used several purine and pyrimidine analogs in growth-based experiments to acquire antimetabolite substrate profiles for these transporters. Overall, our results identify at least one substrate for three out of six NCS1 transporters and seven out of seven NCS2 transporters. Furthermore, our data demonstrate which transporters are functionally important for the uptake of nine nucleobases and four nucleobase analogs. Collectively, these results provide a significant advance in our understanding of nucleobase transport in this clinically relevant organism.

## RESULTS

### Growth-based substrate identification method for NCS1 and NCS2 transporters

We developed a growth-based substrate identification method based on the previously demonstrated ability of pseudomonads to utilize nucleobases as sole nitrogen sources for growth (39, 40). We serially deleted all 13 putative NCS1 and NCS2 transporters from wild type (WT), creating strain Δ*PA1519*Δ*PA4719*Δ*PA1419*Δ*PA0476*Δ*PA5099*Δ*PA2073*Δ*PA4647*Δ*PA0166*Δ*PA0352*Δ*PA2938*Δ*PA1507*Δ*PA0438*Δ*PA0443*, which will subsequently be referred to as Δ13. We then generated pPSV37-based expression vectors for each deleted transporter and used these to individually express transporters in the Δ13 background such that each strain only produced a single NCS1 or NCS2 transporter. We screened the growth of these strains alongside WT and Δ13 with empty pPSV37 vector on M9 media containing 1 of 10 nucleobases as sole nitrogen sources: the purines adenine, hypoxanthine, guanine, xanthine, uric acid, and allantoin or the pyrimidines cytosine, thymine, uracil, and dihydrouracil. We anticipated that we would observe rescue of growth for Δ13 strains expressing a transporter important for nucleobase uptake.

We first confirmed that Δ13 grew similarly to WT under nitrogen-replete conditions (Fig. S1). As expected, Δ13 demonstrated poor growth compared with WT for nearly all nucleobases at their tested concentration, validating the potential use of growth as a phenotypic readout (Fig.S2 and S3). Depending on the substrate, the magnitude of this growth defect ranged from several hours of extension of lag phase to no growth for the duration of the experiment. To quantify growth advantage over Δ13, growth for each replicate was converted to an area under curve (AUC) value, and strains were then compared with statistical analysis. Expression of single NCS1 and NCS2 transporters thus provided initial transporter-substrate identifications.

To validate these observations and to ensure all transporters were identified, we then generated combinatorial transporter deletion strains for each nucleobase substrate. Redundance in transport often required us to make additional deletions to identify the minimum deletion set needed to eliminate transport. We selected these additional transporters based on similarity between *P. aeruginosa* transporters as well as similarity to characterized transporters in other bacteria. We anticipated that the minimum deletion set of transporters would recapitulate the growth defect of Δ13 on the panel of 10 nucleobases containing both purines and pyrimidines. To quantify growth defect relative to WT, growth for each replicate was converted to an AUC value, and strains were then compared with statistical analysis.

### Purine substrate identifications for putative NCS1 and NCS2 transporters

**Fig 1:**
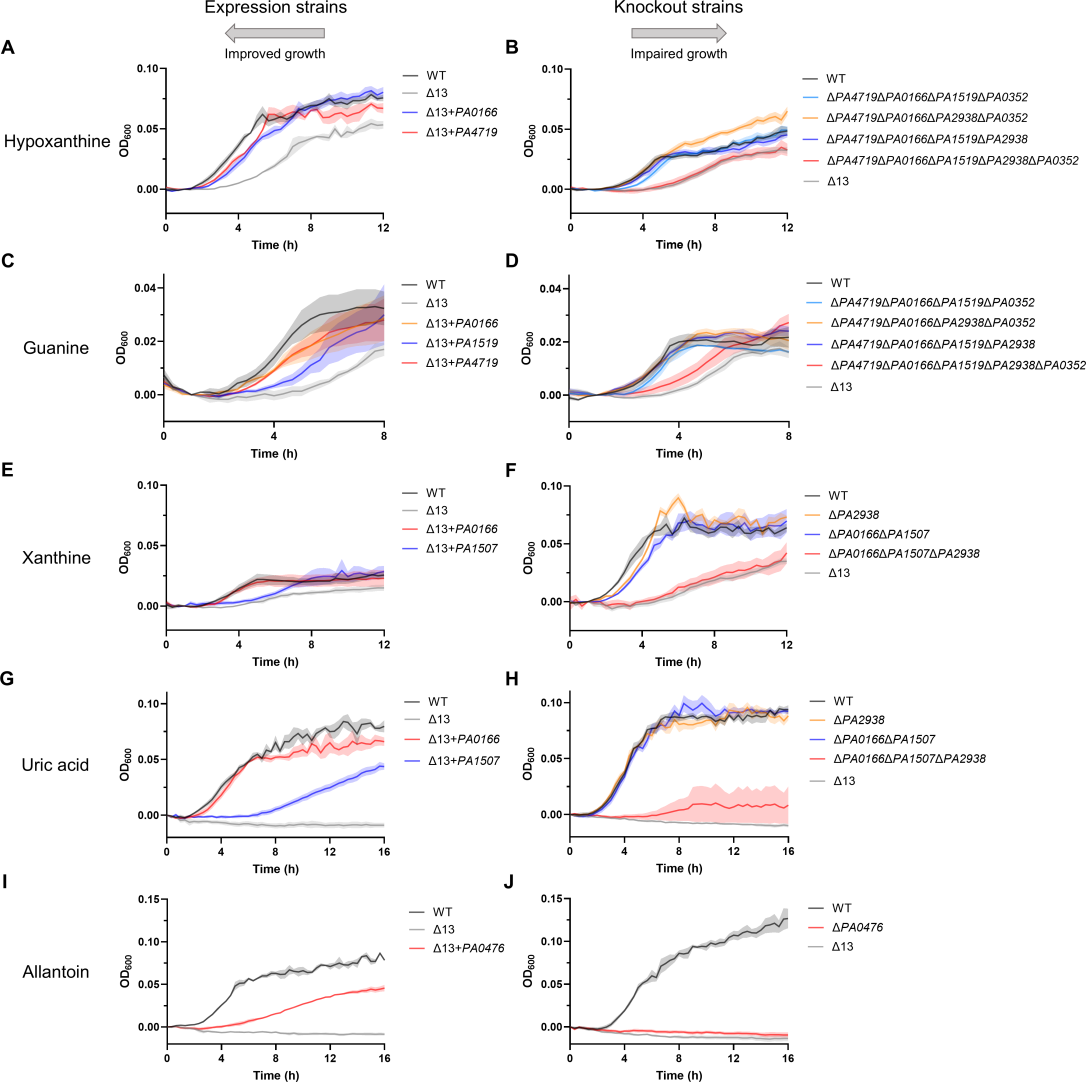
Growth of expression and knockout strains on purines as sole nitrogen sources enabled substrate identification of NCS1 and NCS2 transporters. Growth of selected strains in nitrogen-free M9 supplemented with (**A**) 500-µM hypoxanthine, (**B**) 100-µM hypoxanthine, (**C**) 150-µM guanine, (**D**) 100-µM guanine, (**E**) 300-µM xanthine, (**F**) 300-µM xanthine, (**G**) 300-µM uric acid, (**H**) 300-µM uric acid, (**I**) 500-µM allantoin, or (**J**) 500-µM allantoin. The leftward pointing arrow above the expression strains demonstrates the expectation that expression of relevant transporters will improve growth on a compound as a sole nitrogen source. The rightward pointing arrow above the knockout strains demonstrates the expectation that knocking out relevant transporters will impair growth on a compound as a sole nitrogen source. For each expression strain, one well per substrate per experiment from three independent experiments (*n* = 3) was included. For each knockout strain, one well per substrate per experiment from four independent experiments (*n* = 4) was included. Data represent mean ± SE. For statistical analysis, area under curve values of all strains tested in an experiment were compared using a repeated measures one-way analysis of variance with Dunnett’s multiple comparison test. Strains whose growth significantly differed from the growth of the relevant comparison strain are displayed. Several knockout strains whose growth did not significantly differ from the growth of the relevant comparison strain are also displayed to demonstrate transporter redundancy. Detailed statistical comparisons are available in the main text.

Both hypoxanthine and guanine were associated with PA4719, PA0166, PA1519, PA2938, and PA0352. Specifically, expression strain data supported the importance of PA0166 (*P* < 0.0001) and PA4719 (*P* < 0.0001) for hypoxanthine uptake (Fig. 1A). However, neither single-knockout strain nor Δ*PA4719*Δ*PA0166* grew differently from WT (Fig. S2D). Based on sequence similarity, we suspected that PA0352 (48% similarity to PA0166) and PA1519 (60% similarity to PA4719) may also play a role in hypoxanthine and guanine transport. Growth data for knockout combinations demonstrated that PA0352, PA1519, and PA2938 are important for hypoxanthine uptake in addition to PA0166 and PA4719 (Fig. 1B, *P* < 0.0001). Similarly, expression strain data supported the importance of PA0166 (*P* < 0.0001), PA1519 (*P* < 0.001), and PA4719 (*P* < 0.0001) for guanine uptake (Fig. 1C). However, neither single knockouts nor Δ*PA4719*Δ*PA0166*Δ*PA1519* grew differently from WT (Fig. S2F). As with hypoxanthine, growth data for knockout combinations demonstrated that PA0352 and PA2938 are also important for guanine uptake in addition to PA0166, PA1519, and PA4719 (Fig. 1D, *P* < 0.0001). Consistent with this, PA1519 (39% similarity to EcGhxP) and PA4719 (40% similarity to EcGhxP) are similar to known hypoxanthine-guanine and adenine transporters (6–8). Residual growth of Δ13 on hypoxanthine and guanine suggests that some non-NCS1/NCS2 transporters may also be important for uptake of hypoxanthine and guanine beyond PA4719, PA0166, PA1519, PA2938, and PA0352.

Both xanthine and uric acid were associated with PA0166, PA1507, and PA2938. Specifically, expression strain data supported the importance of PA0166 (*P* < 0.0001) and PA1507 (*P* < 0.0001) for uptake of both xanthine (Fig. 1E) and uric acid (Fig. 1G). This conclusion was supported by further growth rescue at higher induction for PA1507 (Fig. S7 and 8). Expression strain data implied that PA1419 and PA1519 may also contribute to uptake of xanthine and, to a lesser degree, PA0352 for uric acid, but were ruled out due to a lack of clear growth benefit. However, neither single knockout nor Δ*PA1507*Δ*PA0166* grew differently from WT on these substrates (Fig. S2H and J). Based on sequence similarity, we suspected that PA2938 (46% similarity to PA1507) may also play a role in xanthine and uric acid transport. Growth data for knockout combinations demonstrated that PA2938 is important for xanthine and uric acid uptake in addition to PA0166 and PA1507 (Fig. 1F and H, *P* < 0.0001). It is somewhat unexpected that PA1507, PA2938, and PA0166 all transport both xanthine and uric acid since characterized transporters tend to transport either xanthine or uric acid well (5, 12–14). Furthermore, xanthine and uric acid and purines tend to be transported by different transporters than hypoxanthine and guanine for the characterized NCS2 transporters from *B. subtilis* and *E. coli* (5, 12–14). In this regard, PA0166 and PA2938 appear to transport purines regardless of 2-oxo group presence, a seemingly rare capability shared with PlAzg1 and PlAzg2 from the honeybee pathogen *Paenibacillus larvae* (20). Residual growth of Δ13 on xanthine but not uric acid suggests that some non-NCS1/NCS2 transporters may also be important for uptake of xanthine beyond PA0166, PA1507, and PA2938.

Allantoin was associated with PA0476. Specifically, expression strain data implied that PA0476 may be the sole allantoin transporter (Fig. 1I, *P* < 0.0001). This conclusion was supported by further growth rescue at higher induction (Fig. S9). Growth data for Δ*PA0476* suggests PA0476 is the sole allantoin transporter because Δ*PA0476* greatly diminished ability to grow on allantoin (Fig. 1J, *P* < 0.0001). It may be somewhat surprising that PA0476 is not highly similar to known allantoin transporters BsPucI (34% similarity) (4, 5) and EcAllW (31% similarity) (15), but this demonstrates the difficulty in determining substrate specificity from sequence alone.

### Pyrimidine substrate identifications for putative NCS1 and NCS2 transporters

**Fig 2:**
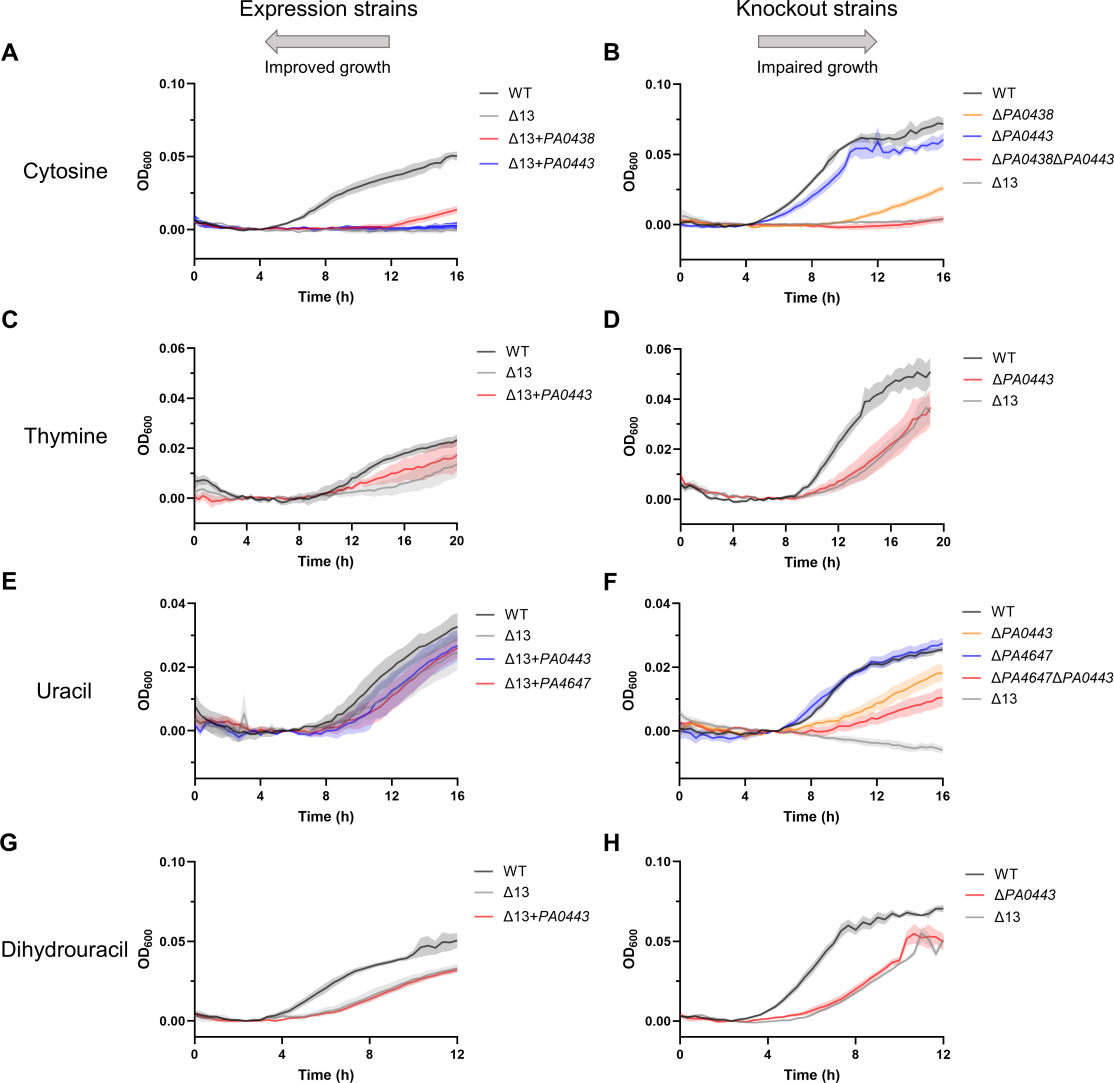
Growth of expression and knockout strains on pyrimidines as sole nitrogen sources enabled substrate identification of NCS1 and NCS2 transporters. Growth of selected expression and knockout strains in nitrogen-free M9 supplemented with (**A**) 500-µM cytosine, (**B**) 500-µM cytosine, (**C**) 500-µM thymine, (**D**) 500-µM thymine, (**E**) 500-µM uracil, (**F**) 100-µM uracil, (**G**) 500-µM dihydrouracil, or (**H**) 500-µM dihydrouracil. The leftward pointing arrow above the expression strains demonstrates the expectation that expression of relevant transporters will improve growth on a compound as a sole nitrogen source. The rightward pointing arrow above the knockout strains demonstrates the expectation that knocking out relevant transporters will impair growth on a compound as a sole nitrogen source. For each expression strain, one well per substrate per experiment from three independent experiments (*n* = 3) was included. For each knockout strain, one well per substrate per experiment from four independent experiments (*n* = 4) was included. Data represent mean ± SE. For statistical analysis, area under curve values of all strains tested in an experiment were compared using a repeated measures one-way analysis of variance with Dunnett’s multiple comparison test. Strains whose growth significantly differed from the growth of the relevant comparison strain are displayed. Expression strain Δ13 + *PA0443* did not grow significantly different from Δ13 in cytosine, uracil, and dihydrouracil conditions nor did Δ13 + *PA4647* grow significantly different from Δ13 in the uracil condition. Several knockout strains whose growth did not significantly differ from the growth of the relevant comparison strain are also displayed to demonstrate transporter redundancy. Detailed statistical comparisons are available in the main text.

Cytosine was associated with PA0438 and PA0443. Expression strain data implied that PA0438 may be the sole cytosine transporter (Fig. 2A, *P* < 0.0001). However, the lack of complete growth rescue with PA0438 implied that another transporter may also contribute to cytosine uptake. Indeed, the defect in growth for Δ*PA0438* was not as large as that of Δ13 (Fig. 2B, *P* < 0.0001). Based on genomic location near *PA0438* and genes predicted to be involved in pyrimidine catabolism, we hypothesized PA0443 may also be involved in cytosine uptake. While Δ*PA0443* alone did not have a notable growth defect on cytosine, Δ*PA0438*Δ*PA0443* had a significant growth defect compared to Δ*PA0438* (Fig. 2B, *P* < 0.0001). Consistent with this, PA0438 is similar to EcCodB (75% similarity), which has been previously demonstrated to transport cytosine (3). Apparent residual growth of Δ13 on cytosine suggests that some non-NCS1/NCS2 transporters may also play a minor role in uptake of cytosine beyond PA0438 and PA0443.

Thymine was associated with PA0443. Specifically, expression strain data implied that PA0443 may be the sole NCS1/NCS2 thymine transporter (Fig. 2C, *P* < 0.0001). Indeed, Δ*PA0443* displayed a significant growth defect relative to WT (*P* < 0.0001) comparable to Δ13, suggesting that PA0443 may be the sole NCS1/NCS2 transporter for thymine (Fig. 2D). Residual growth of Δ13 on thymine suggests that some non-NCS1/NCS2 transporters may also be important for thymine uptake beyond PA0443.

Uracil was associated with PA4647 and PA0443. Expression strain data for growth on uracil were inconclusive regarding which transporters may be important for uptake (Fig. 2E), so we tested combinatorial deletion strains at a lower uracil concentration where a lack of high-affinity transport would be more apparent. Knockout strain data showed that Δ*PA0443* (*P* < 0.0001), but neither Δ*PA0438* nor Δ*PA4647*, had a growth defect on uracil compared to WT (Fig. 2F; Fig. S3F). However, the growth defect for Δ*PA0443* was not as severe as that of Δ13. We suspected PA4647 may also play a role in uracil transport based on similarity to EcRutG (39% similarity) (9, 10) and EcUraA (39% similarity) (11), both of which are known uracil transporters. As expected, the combination knockout Δ*PA4647*Δ*PA0443* grew worse than Δ*PA0443* (*P* < 0.0001), suggesting that PA0443 and PA4647 both contribute to uracil uptake (Fig. 2F). The strong growth of Δ13 on higher uracil concentrations suggests the presence of a lower-affinity non-NCS1/NCS2 uracil transporter beyond PA4647 and PA0443.

Dihydrouracil was associated with PA0443. Expression strain data for growth on dihydrouracil were also inconclusive regarding which transporters may be important for uptake (Fig. 2G), so we again tested deletion strains. Knockout strain data showed that Δ*PA0443* (*P* < 0.0001), but neither Δ*PA0438* nor Δ*PA4647*, had a growth defect on dihydrouracil compared to WT (Fig. 2H; Fig. S3H). Indeed, Δ*PA0443* displayed a significant growth defect relative to WT (*P* < 0.0001) comparable to Δ13, suggesting that PA0443 may be the sole NCS1/NCS2 transporter for dihydrouracil (Fig. 2H). Residual growth of Δ13 suggests that some non-NCS1/NCS2 transporters may also be important for dihydrouracil uptake beyond PA0443.

### Antimetabolite substrate identifications for putative NCS1 and NCS2 transporters

We next characterized these transporters using toxic analogs of the previously tested nucleobases known as antimetabolites. Antimetabolites not only provide additional support for the identified transporters but also to lend insight into which transporters may be important for antimetabolite therapy. In contrast to the expression strains, we anticipated that we would observe growth defects for Δ13 strains expressing a transporter important for antimetabolite uptake. We grew WT and Δ13 control strains with empty pPSV37 vector and Δ13 expression strains on M9 with sufficient nitrogen for robust growth, which had been supplemented with one of four antimetabolites: the purine analogs 8-azaguanine and 6-thioguanine or the pyrimidine analogs 5-fluorocytosine and 5-fluorouracil. As expected, Δ13 grew better than WT on all antimetabolites at their tested concentration (Fig. S4, *P* < 0.0001). Depending on the antimetabolite, the magnitude of improved growth for Δ13 ranged from several hours to approximately 14 hours.

**Fig 3:**
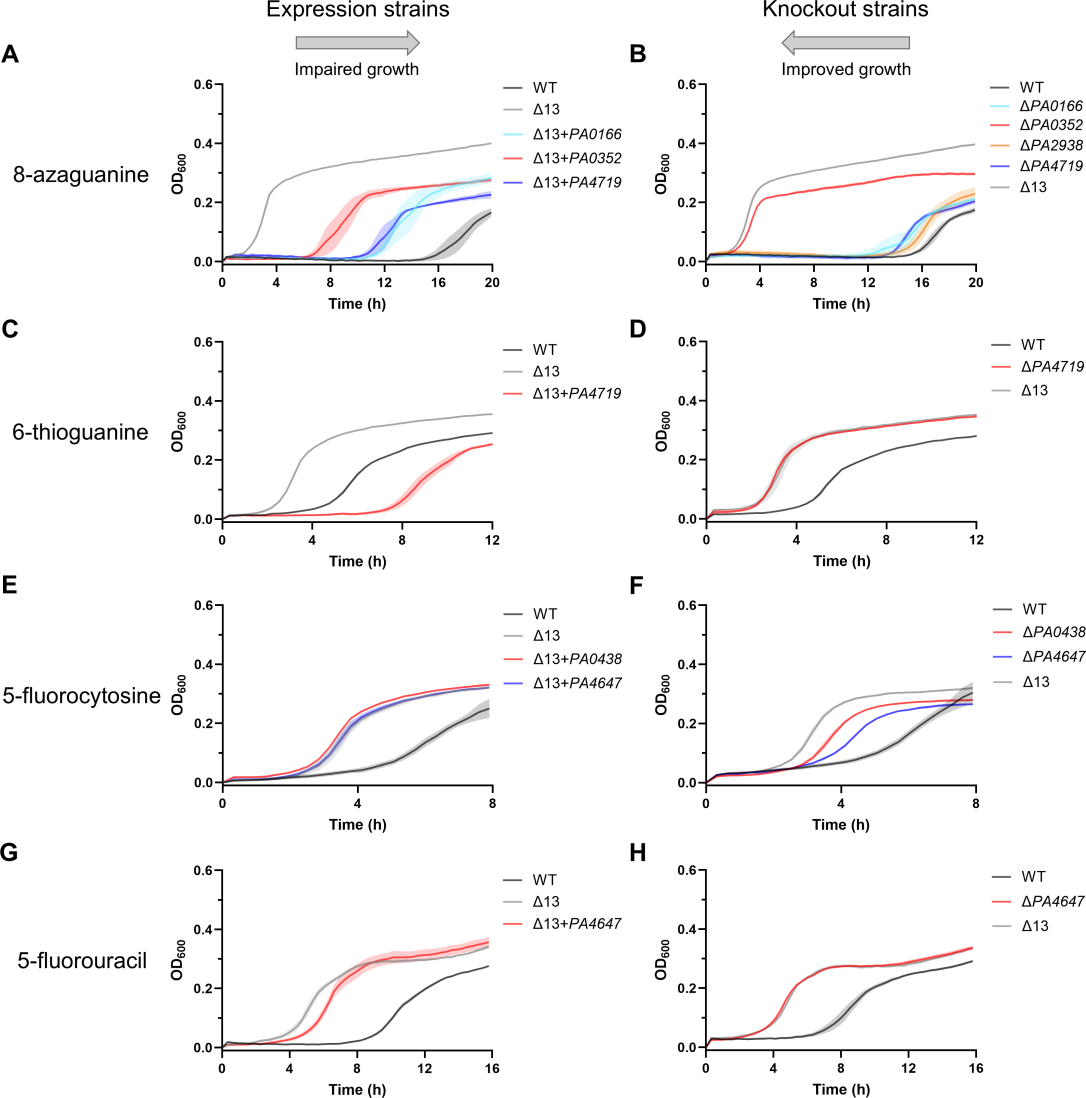
Growth of expression and knockout strains on nitrogen-replete media exposed to toxic nucleobase analogs enabled substrate identification of NCS1 and NCS2 transporters. Growth of selected expression and knockout strains in M9 containing casamino acids and the toxic compounds (**A**) 1,000-µM 8-azaguanine, (**B**) 1,000-µM 8-azaguanine, (**C**) 100-µM 6-thioguanine, (**D**) 100-µM 6-thioguanine, (**E**) 1,000-µM 5-fluorocytosine, (**F**) 1,000-µM 5-fluorocytosine, (**G**) 10-µM 5-fluorouracil, and (**H**) 10-µM 5-fluorouracil. The rightward pointing arrow above the expression strains demonstrates the expectation that expression of relevant transporters will impair growth when exposed to a toxic nucleobase analog. The leftward pointing arrow above the knockout strains demonstrates the expectation that knocking out relevant transporters will improve growth when exposed to a toxic nucleobase analog. For each expression strain, one well per substrate per experiment from three independent experiments (*n* = 3) was included. For each knockout strain, one well per substrate per experiment from four independent experiments (*n* = 4) was included unless otherwise stated due to removal of outliers. For 6-thioguanine knockout experiments, *n* = 3 for WT and Δ*PA4719* and *n* = 2 for Δ13. For 5-fluorocytosine knockout experiments, *n* = 2 for Δ*PA4647* and Δ13. For 5-fluorouracil knockout experiments, *n* = 2 for Δ13. Data represent mean ± SE. For statistical analysis, area under curve values of all strains tested in an experiment were compared using a repeated measures one-way analysis of variance with Dunnett’s multiple comparison test. Strains whose growth significantly differed from the growth of the relevant comparison strain are displayed. Detailed statistical comparisons are available in the main text.

Similar to guanine, 8-azaguanine was associated with PA4719, PA0166, PA1519, PA2938, and PA0352. Specifically, expression strain data implied that PA0352 (*P* < 0.0001) and, to a somewhat greater extent, PA0166 (*P* < 0.0001) and PA4719 (*P* < 0.0001) caused the lag phase to increase by approximately 6–10 hours, suggesting that they all contributed to 8-azaguanine uptake (Fig. 3A). It is not possible to immediately determine from these data whether PA0166 and PA4719 are more important than PA0352 for transport of 8-azaguanine. Surprisingly, Δ*PA0352* grew significantly better than WT (*P* < 0.0001) comparable to Δ13, suggesting that PA0352 may be the primary transporter for 8-azaguanine (Fig. 3B). Δ*PA0166* (*P* < 0.0001), Δ*PA2938* (*P* < 0.0001), and Δ*PA4719* (*P* < 0.0001) grew better than WT, suggesting they also likely contribute to 8-azaguanine uptake (Fig. 3B). No other single-knockout strain grew substantially better than WT when exposed to 8-azaguanine (Figure S4B).

6-Thioguanine was associated with PA4719. Specifically, expression of PA4719 caused the lag phase to increase by approximately 6 hours (Fig. 3C, *P* < 0.0001). Intriguingly, expression strain data for PA4719 are more severe than that of WT, implying that PA4719 may be expressed more highly in this strain than in WT (Fig. 3C). As expected, Δ*PA4719* grew significantly better than WT (*P* < 0.0001) comparable to Δ13, suggesting that PA4719 may be the primary transporter for 6-thioguanine (Fig. 3D). No other single-knockout strain grew substantially better than WT when exposed to 6-thioguanine (Fig. S4D).

5-Fluorocytosine and 5-fluorouracil were associated with PA4647. All Δ13 expression vector strains grew similarly to Δ13, implying that whichever transporter or transporters are important for the uptake of these antimetabolites may not be properly expressed in these strains (Fig. 3E and G; Fig.S4E through G). However, Δ*PA0438* (*P* < 0.0001) and Δ*PA4647* (*P* < 0.0001) both grew better than WT, suggesting that PA0438 and PA4647 contribute to 5-fluorocytosine uptake (Fig. 3F). No other single-knockout strain grew substantially better than WT when exposed to 5-fluorocytosine (Fig. S4F). As expected, Δ*PA4647* grew significantly better than WT (*P* < 0.0001) comparable to Δ13, suggesting that PA4647 may be the primary transporter for 5-fluorouracil (Fig. 3H). No other single-knockout strain grew substantially better than WT when exposed to 5-fluorouracil (Figure S4H).

### Phylogenetic tree summarizing NCS1 and NCS2 transporter-substrate identifications

**Fig 4:**
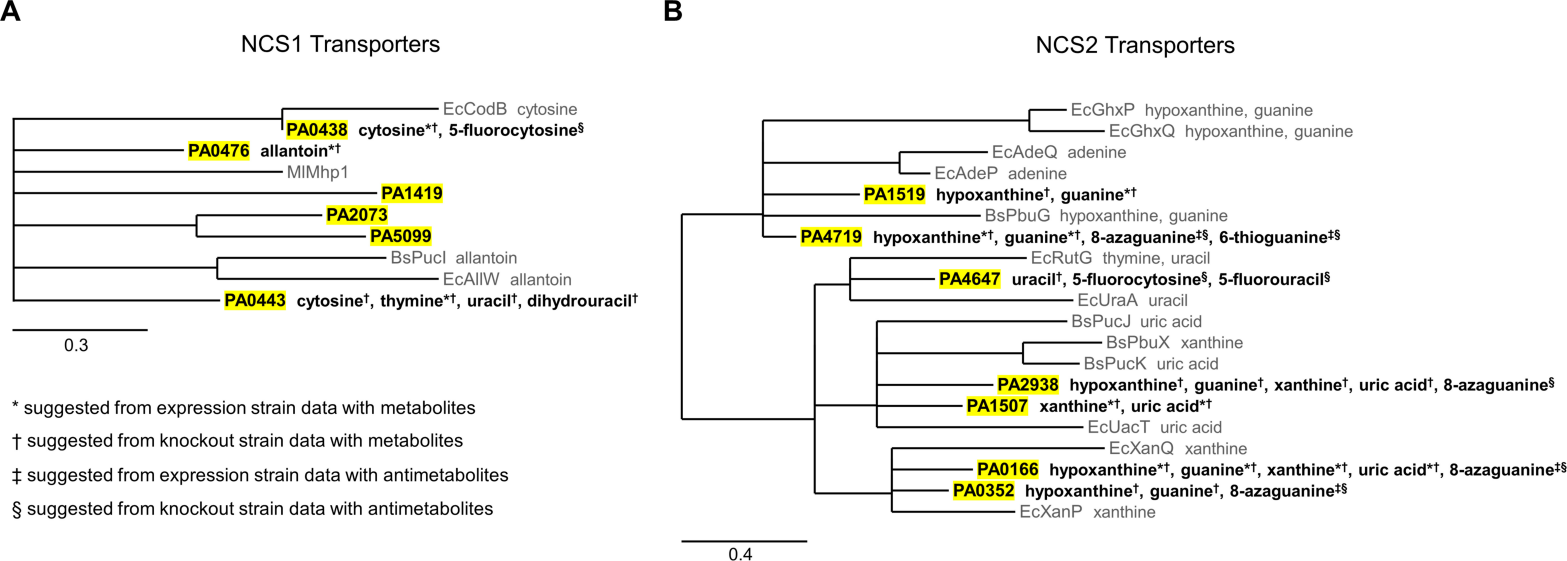
Summary of data in the context of NCS1 and NCS2 phylogenetic trees. (**A**) Maximum-likelihood phylogenetic trees depict *P. aeruginosa* transporters and selected characterized bacterial NCS1 and (**B**) NCS2 transporters based on nucleotide sequences. Branches with bootstrap support lower than 70% were collapsed. Substrate specificity for characterized transporters is shown after transporter name. *P. aeruginosa* transporter names are highlighted, and substrate specificity for these transporters are also shown after transporter name. For the substrates after *P. aeruginosa* transporters, * indicates this is suggested by expression strain data with metabolites; † indicates this is suggested by knockout strain data with metabolites; ‡ indicates this is suggested by expression strain data with antimetabolites; and § indicates this is suggested by knockout strain data with antimetabolites. Scale bar indicates the number of substitutions per site.

To summarize all transporter-substrate identifications, we generated a maximum-likelihood phylogenetic tree using Phylogeny.fr (41–46) for characterized NCS1 transporters from *E. coli*, *B. subtilis*, and *Microbacterium liquefaciens*, along with the six putative NCS1 transporters from *P. aeruginosa* (Fig. 4A). This phylogenetic tree represents all the characterized bacterial NCS1 transporters (3–5, 15, 47), demonstrating how little is known about bacterial NCS1 transporters to date. We also generated a maximum-likelihood phylogenetic tree using Phylogeny.fr (41–46) for characterized NCS2 transporters from *E. coli* and *B. subtilis*, along with the seven putative NCS2 transporters from *P. aeruginosa* (Fig. 4B). This phylogenetic tree represents all the characterized NCS2 transporters from the most studied bacteria, such as *E. coli* and *B. subtilis*, but not necessarily all characterized bacterial NCS2 transporters (5–14). Our experimental evidence does not suggest which substrates are transported by PA1419, PA2073, or PA5099 (Fig.S2 through S4). This lack of substrate identification may be due either to low native expression, low transport affinity, or transport of a substrate beyond those tested here.

## DISCUSSION

Our results provide initial substrate identifications for 10 putative nucleobase transporters in *P. aeruginosa*. Many of our substrate assignments are supported by data from multiple distinct experiments. For example, types of experiments tested include rescue of growth for transporters on expression vectors vs. decreased growth for knockout strains, as well as testing for growth on nucleobases as a sole nitrogen source vs. growth in presence of toxic nucleobase analogs. Our data are further validated by phylogenetic clustering of transporters with similar substrate profiles between the NCS1 and NCS2 transporters characterized in this work and previously characterized bacterial transporters. Apparent functional redundancy of transport suggests that growth phenotypes are not caused by an indirect or polar effect in a particular genomic neighborhood. The ability to closely recapitulate growth phenotypes observed for the Δ13 with separately generated knockout strains also strengthens our conclusions. Nevertheless, transporters capable of transporting a particular nucleobase may fail to display a phenotype in these experiments if the transporter is not expressed at a sufficient level in tested conditions or if the capacity or affinity of the transporter is too low to affect growth. Thus, while these data suggest which transporters are important for the uptake of 10 nucleobases and 4 nucleobase analogs, it is possible that some tested substrates are transported by NCS1 and NCS2 transporters not associated with a growth phenotype in this work. In the future, radiolabeled substrate experiments may unveil additional substrate-transporter pairs not able to be detected by a growth phenotype. These radiolabeled experiments could also quantify transport kinetics and substrate affinity for these NCS1 and NCS2 transporters, which cannot be determined from these growth experiments.

In contrast to the redundancy in uptake we observed for most nucleobases, single-transporter knockout strains exhibited resistance to tested nucleobase analogs. Specifically, PA0352 is important for 8-azaguanine resistance; PA4719 is important for 6-thioguanine resistance; PA0438 and PA4647 are important for 5-fluorocytosine resistance;, and PA4647 is important for 5-fluorouracil resistance. Therefore, the loss of different single transporters appears to be important for resistance to each toxic nucleobase analog. Since single enzymes (33, 34) also tend to be important for the activation of these antimetabolites, combination therapy of at least two distinct antimetabolites—or an antimetabolite and another antimicrobial agent—may be recommended to reduce development of resistance to these compounds.

The widespread capability among organisms to transport and catabolize nucleobases also highlights the importance of nucleobases as a potential nutrient source (28). Adenine is present in seawater albeit at a low level (48) while pyrimidines are likely available in pond and tap water (49). In fact, a TnSeq experiment suggests that PA0443, which we suspect to be a pyrimidine transporter with broad specificity, may be important for growth in pond and tap water (49). In terrestrial environments, nucleobases and related molecules may represent up to 7% of total available nitrogen in the soil and up to nearly 20% of available nitrogen in humus, a soil component (50). Localized areas in the soil can be further enriched in xanthine due to stress-induced release of metabolites by the roots of plants, which can selectively increase the abundance of plant-associated *Pseudomonas* spp. (51). In this environment, PA0166, PA1507, and PA2938, which we identify as xanthine transporters, may be relevant. Localized areas in the soil can also be enriched in nucleobases by animal waste products (52), which are rich in nucleobases (53, 54). Thus, nucleobases are available to various degrees in different environments that *P. aeruginosa* may encounter.

In the context of infection, while nucleobases are generally limited at many body sites, certain nucleobases may accumulate to a sufficiently high concentration to alter growth. Sputum from patients with cystic fibrosis (CF) (55) and metabolic cross-feeding between polymicrobial communities in CF lung infection (27) can both support growth of purine auxotrophic strains, suggesting purines may be available in this environment. Work with *Klebsiella pneumoniae* suggests that allantoin may be available in the liver (56). If this is also true in *P. aeruginosa* pathogenesis, PA0476, as the sole allantoin transporter, may be important in liver infections. While serum allantoin levels may be relatively low at approximately one-tenth to one-twentieth of the concentration used in our experiments, serum uric acid levels in healthy individuals can approach and even exceed the 300-µM uric acid used in our experiments (57). Uric acid concentrations in synovial fluids and urine are 200 µM (57) and 2 mM (58), respectively. These values are all sufficient to support *P. aeruginosa* growth as sole nitrogen sources. Thus, PA0166, PA1507, and PA2938 could contribute to growth in several body fluids. In addition to specific body sites, *P. aeruginosa* that has invaded host cells (59, 60) may be able to access relatively high levels of intracellular hypoxanthine and guanine (61). Alternatively, *P. aeruginosa* may be able to utilize these nucleobases when cytoplasmic contents leak from dying or otherwise damaged tissues (62–65). In these situations, some combination of PA0166, PA0352, PA1519, PA2938, and PA4719 may be important since our data suggest they are hypoxanthine-guanine transporters. The degree of redundancy for transport of hypoxanthine and guanine suggests these molecules may be especially important to *P. aeruginosa*, a conclusion which is further supported by recent discovery of a hypoxanthine-guanine-specific chemoreceptor for *P. aeruginosa* chemotaxis (66).

Beyond their role as a nutrient source, nucleobases and related molecules may additionally act as a cue to alter bacterial behavior. Several second messenger signaling molecules in bacteria, such as cAMP (67), c-di-AMP (68, 69), c-di-GMP (70), and (*P*)ppGpp (71, 72), are generated from purine nucleotide substrates. Purine nucleotide pools, which can be affected by the salvage of exogenous nucleobases (73–77), may represent a regulatory point for nucleotide-derived second messengers (78). Indeed, adenine and guanine have been shown to alter *Staphylococcus aureus* susceptibility to antimicrobial treatment by changing c-di-AMP levels (79), while adenine and hypoxanthine have been shown to decrease *P. aeruginosa* biofilm formation by decreasing c-di-GMP levels (80). Since these molecules must be salvaged from the environment to affect nucleotide pools in this manner (79, 80), NCS1 and NCS2 transporters may be critical to the signal transduction of nucleobase signaling. However, these transporters are unlikely to be identified as hits in forward genetic screens even in situations where nucleobase transport is critical due to redundancy in uptake for most native substrates. Therefore, strains generated in this work may be useful tools to address previously difficult-to-test hypotheses relating to nucleobase transport.

## MATERIALS AND METHODS

### Bacterial strains and growth conditions

The full strain list is available in File S1. *P. aeruginosa* MPAO1 was obtained from the University of Washington (81). Bacteria were frozen in 50% glycerol-50% Luria-Bertani (LB) media (Fisher Bioreagents). Solid media were prepared by adding 1.5-g/L agar (Fisher Bioreagents) to liquid media before autoclaving. *E. coli* and *P. aeruginosa* strains were routinely grown on LB agar at 37°C overnight, and single colonies were used to innoculate LB media for growth at 37°C overnight with shaking at 250 rpm unless otherwise stated. When appropriate, 50 µg/mL (*E. coli*) or 250 µg/mL (*P. aeruginosa*) carbenicillin (Sigma-Aldrich), 15 µg/mL (*E. coli*) or 50 µg/mL (*P. aeruginosa*) gentamicin (TCI), or 5 µg/mL (*P. aeruginosa*) irgasan (Sigma-Aldrich) was added to media for selection. Sucrose (7.5%, Sigma-Aldrich) was added to no-salt LB agar—10-g/L tryptone (Fisher Bioreagents) and 5-g/L yeast extract (Fisher Bioreagents)—for sucrose counterselection. For use in experiments, *P. aeruginosa* was grown overnight in M9 media. Nitrogen-free M9 media for wash steps contained 47.7-mM Na_2_HPO_4_ (Sigma-Aldrich), 21.7-mM KH_2_PO_4_ (Sigma-Aldrich), 8.6-mM NaCl (Sigma-Aldrich), 0.2% glucose (Sigma-Aldrich), and 1-mM MgSO_4_ (Sigma-Aldrich) and were supplemented with 18.7-mM NH_4_Cl (Fisher Chemical) and 0.5% acid casein peptone (Fisher Bioreagents) for overnight growth. M9 media for nucleobase growth experiments contained 39.7-mM Na_2_HPO_4_ (Sigma-Aldrich), 18.1-mM KH_2_PO_4_ (Sigma-Aldrich), 7.2-mM NaCl (Sigma-Aldrich), 0.2% glucose (Sigma-Aldrich), and 1-mM MgSO_4_ (Sigma-Aldrich) and were supplemented with 15.6-mM NH_4_Cl (Fisher Chemical) and 0.5% acid casein peptone (Fisher Bioreagents) for nucleobase analog growth experiments.

### Generation of knockout strains

Allelic exchange was used to generate in-frame knockout strains of *P. aeruginosa* MPAO1 (82). Briefly, regions upstream and downstream of genes of interest were amplified from the MPAO1 genome by PCR including Gibson overhangs using Phusion Green Hot Start II High-Fidelity PCR Master Mix (New England BioLabs). The primer list is available in File S2. The plasmid list is available in File S3. pEXG2 vector was digested with HinDIII-HF (New England BioLabs) (83). Upstream and downstream regions were combined in three-part Gibson assembly with cut pEXG2 using Gibson Assembly Master Mix (New England BioLabs). NEB 5-alpha Competent *E. coli* (New England BioLabs) was chemically transformed with constructs, and insert presence was detected by PCR and verified by Sanger sequencing. *E. coli* S17-1 (84) was chemically transformed with verified constructs. Constructs were then mated into *P. aeruginosa* by conjugation. Sucrose counterselection was used to resolve merodiploids. Single-knockout strains were verified by amplifying regions of interest by PCR and sequencing with Sanger sequencing. Combination knockout mutants were generated using the same process with a single knockout or combination knockout mutant as the recipient strain of the pEXG2 deletion construct instead of MPAO1. Subsequent combination mutants were verified by amplifying regions of interest by PCR.

### Generation of expression vectors

NCS1 and NCS2 transporters were expressed using the expression vector pPSV37 (85). Briefly, genes of interest were amplified from the MPAO1 genome by PCR including Gibson overhangs using Phusion Green Hot Start II High-Fidelity PCR Master Mix (New England BioLabs) or KOD One PCR Master Mix Blue (Toyobo). pPSV37 vector was digested with HinDIII-HF (New England BioLabs). The genes were combined in two-part Gibson assembly with cut pPSV37 using Gibson Assembly Master Mix (New England BioLabs). NEB 5-alpha Competent *E. coli* (New England BioLabs) was chemically transformed with constructs, and insert presence was detected by PCR and verified by Sanger sequencing and/or Nanopore sequencing. *P. aeruginosa* Δ13 was then chemically transformed with verified constructs to generate Δ13 + pPSV37 expression vector strains. MPAO1 and Δ13 were chemically transformed with empty pPSV37 expression vectors for use as control strains.

### Nucleobase and nucleobase analog chemicals

Stock compounds of adenine (Alfa Aesar), hypoxanthine (Acros Organics), guanine (Acros Organics), xanthine (Acros Organics), uric acid (Alfa Aesar), allantoin (TCI), cytosine (TCI), thymine (Acros Organics), uracil (Acros Organics), 5,6-dihydrouracil (Alfa Aesar), 8-azaguanine (J&K Scientific), 6-thioguanine (Alfa Aesar), 5-fluorocytosine (TCI), and 5-fluorouracil (TCI) were created by dissolving compounds in water. Compounds that did not readily dissolve were rotated on a Tube Revolver Rotator (Thermo Scientific), heated in a 55°C water bath, and/or vortexed until solubilized.

### Growth experiments with nucleobase metabolites

For transporter expression growth experiments, indicated strains were grown overnight in M9 supplemented with gentamicin and 1-mM isopropyl-β-D-thiogalactopyranoside (IPTG). Strains were pelleted via centrifugation, resuspended in nitrogen-free M9, pelleted via centrifugation, and resuspended in nitrogen-free M9 a second time to wash away residual nitrogen-containing components in overnight M9 media. Two-microliters washed overnight culture of each strain was added to 198-µL nitrogen-free M9 supplemented with an individual nucleobase in a clear 96-well plate (Nunc, Thermo Scientific) at specified final concentrations; wells were covered with Breathe-Easy film (USA Scientific); and the plate was shaken at 37°C in a Synergy Neo2 plate reader with an absorbance measurement at 600 nm taken every 20 minutes for at least 16 hours. Initial OD_600_ for each well was subtracted from all time points of that well as background except for the following substrates whose background was instead taken as the time point indicated in parentheses—hypoxanthine, guanine, and xanthine (1 hour), cytosine (4 hours), thymine (6 hours), uracil (5.7 hours), and dihydrouracil (2.3 hours)—to improve synchronicity of growth readings. Three independent experiments were performed. Plots were generated using GraphPad Prism.

For knockout growth experiments, indicated strains were grown overnight in M9, and the same protocol was followed as for transporter expression experiments. Initial OD_600_ for each well was subtracted from all time points of that well as background except the following substrates whose background was instead taken as the time point indicated in parentheses—hypoxanthine, guanine, and xanthine (1 hour), cytosine (4 hours), thymine (6 hours), uracil (5.7 hours), and dihydrouracil (2.3 hours)—to improve synchronicity of growth readings. Four independent experiments were performed. Plots were generated using GraphPad Prism.

### Growth experiments with nucleobase analog antimetabolites

For transporter expression growth experiments, indicated strains were grown overnight supplemented with gentamicin and 1-mM IPTG. Strains were pelleted via centrifugation, resuspended in nitrogen-free M9, pelleted via centrifugation, and resuspended in nitrogen-free M9 a second time to wash away residual nitrogen-containing components in overnight M9 media. Two-microliters washed overnight culture of each strain was added to 198-µL full M9 supplemented with an individual nucleobase analog antimetabolite in a clear 96-well plate (Nunc, Thermo Scientific) at specified final concentrations; wells were covered with Breathe-Easy film (USA Scientific); and the plate was shaken at 37°C in a Tecan Infinite MPlex plate reader with an absorbance measurement at 600 nm taken every 20 minutes for at least 16 hours. Initial OD_600_ for each well was subtracted from all time points of that well as background to improve synchronicity of growth readings. Three independent experiments were performed. Plots were generated using GraphPad Prism.

For knockout growth experiments, indicated strains were grown overnight in M9, and the same protocol was followed as for transporter expression growth experiments with antimetabolites. Initial OD_600_ for each well was again subtracted from all time points of that well as background to improve synchronicity of growth readings. Four independent experiments were performed. Plots were generated using GraphPad Prism.

### Comparison of protein sequence identity

Amino acid sequences for putative *P. aeruginosa* NCS1 and NCS2 transporters in strain PAO1 were downloaded from the Pseudomonas Genome Database via Pseudomonas.com (38). Amino acid sequences for characterized *E. coli* NCS1 and NCS2 transporters in strain K-12 MG1655 were downloaded from the BioCyc Database Collection via BioCyc.org (86). Pairwise amino acid sequences were compared with blastp using default parameters, and the resulting amino acid percent identity was rounded to the nearest integer (87).

### Generation of phylogenetic trees

Nucleotide sequences for putative *P. aeruginosa* NCS1 and NCS2 transporters in strain PAO1 were downloaded from the Pseudomonas Genome Database via Pseudomonas.com (38). Nucleotide sequences for characterized *E. coli* NCS1 and NCS2 transporters in strain K-12 MG1655 were downloaded from the BioCyc Database Collection via BioCyc.org (86). Nucleotide sequences for characterized *B. subtilis* NCS1 and NCS2 transporter nucleotide sequences in strain 168 were downloaded from Subtiwiki.com (88). Nucleotide sequence for characterized NCS1 transporter Mhp1 in strain *M. liquefaciens* AJ 3912 was extracted from Suzuki et al. (89). Multiple nucleotide sequences were aligned with MUSCLE (41); alignments were curated with Gblocks (42); phylogenetic trees were reconstructed with PhyML (43); and trees were visualized with TreeDyn (44) as packaged in phylogeny.fr (45). Five hundred bootstraps were used for internal branch reliability (46). Branches with bootstrap support lower than 70% were collapsed. *P. aeruginosa* protein names were highlighted, and transporter-substrate assignments were added after protein names based on prior literature and our experimental data.

### Quantification and statistical analysis

Growth data for each replicate were converted to an AUC value using GraphPad Prism, and strains were then compared to the relevant comparison strain with statistical analysis. Growth was defined as positive peaks starting at the time point used to remove background for each substrate and ending at the last displayed time point in each graph. Data from relevant supplementary figures were used for analyses so that all tested strains were included. Repeated measures one-way analysis of variance with Dunnett’s multiple comparison test comparing WT, Δ13, or another strain, as appropriate, was used to determine statistical significance. Expression and knockout strains were considered statistically different from their comparison strain when *P* < 0.01 and strain AUC was at least 25% greater or at least 25% less than their comparison strain, depending on the comparison being made. Analyses were performed using GraphPad Prism v.10.2.2.

## SUPPLEMENTAL MATERIAL

File S1 mbio.02434-24-s0001.xlsx Strains used in the study.

File S2 mbio.02434-24-s0002.xlsx Primers used in the study.

File S3 mbio.02434-24-s0003.xlsx Plasmids used in the study.

Supplemental Figures mbio.02434-24-s0004.docx Figures S1 to S13.

ASM does not own the copyrights to Supplemental Material that may be linked to, or accessed through, an article. The authors have granted ASM a non-exclusive, world-wide license to publish the Supplemental Material files. Please contact the corresponding author directly for reuse.
