# Supplementary material for: Substrate identification of putative NCS1 and NCS2 nucleobase transporters in Pseudomonas aeruginosa
Source: mBio. 2024 Oct 30;15(12):e02434-24. doi: 10.1128/mbio.02434-24 (PMC11633122; doi:10.1128/mbio.02434-24)
Supplement: Supplemental Figures — Figures S1 to S13. [file mbio.02434-24-s0004.docx]

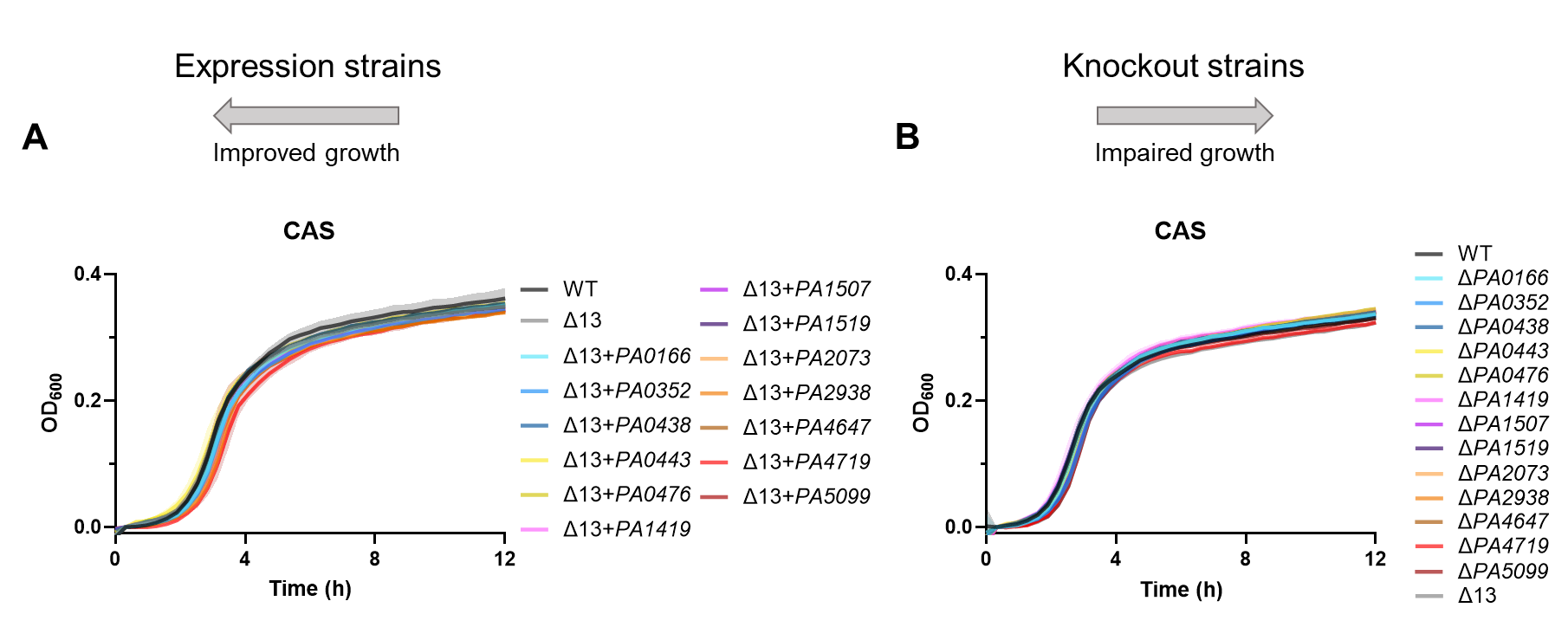


**Supplementary Figure 1. Growth of expression and knockout strains in nitrogen-replete media.** Growth of selected **(A)** expression strains and **(B)** knockout strains in M9 containing casamino acids. The leftward pointing arrow above the expression strains demonstrates the expectation that expression of relevant transporters will improve growth on a compound as a sole nitrogen source. The rightward pointing arrow above the knockout strains demonstrates the expectation that knocking out relevant transporters will impair growth on a compound as a sole nitrogen source. For each expression strain, 1 well per substrate per experiment from 3 independent experiments (n=3) was included unless otherwise stated due to removal of outliers. For expression strains, n=2 for PA0438, PA1507, PA1519, and PA2073. For each knockout strain, 1 well per substrate per experiment from 4 independent experiments (n=4) was included unless otherwise stated due to removal of outliers. For knockout strains, n=3 for Δ*PA0352*, Δ*PA1519*, and Δ*PA2073* and n=2 for Δ*PA4719*. Data represent mean ± SE. For statistical analysis, area under curve (AUC) values of all strains tested in an experiment were compared using a repeated measures one-way ANOVA with Dunnett’s multiple comparison test. Detailed statistical comparisons are available in the main text.


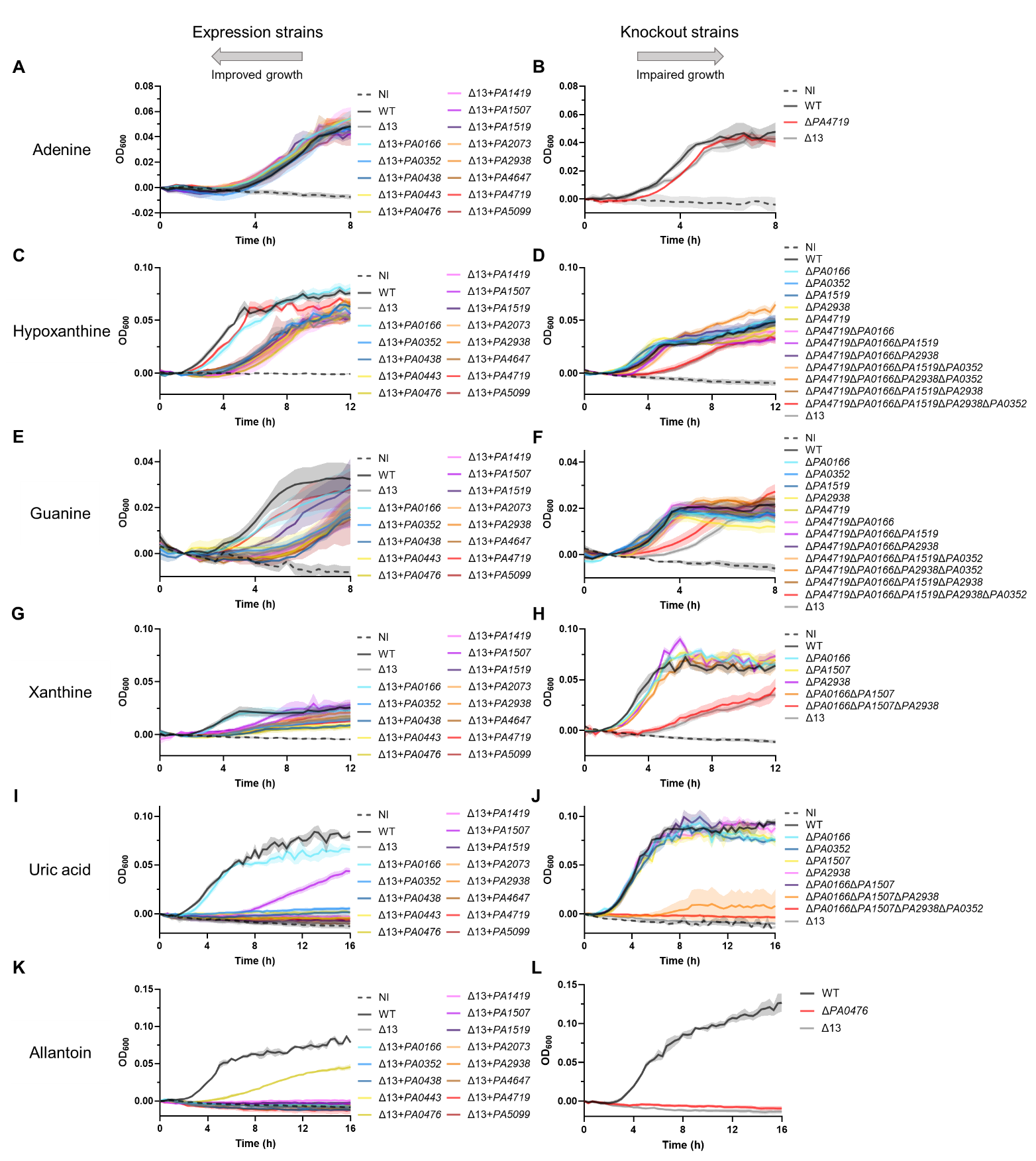


**Supplementary Figure 2. Growth of expression and knockout strains on purines as sole nitrogen sources enabled substrate identification of NCS1 and NCS2 transporters.** Growth of selected strains in nitrogen-free M9 supplemented with **(A)** 500 μM adenine, **(B)** 500 μM adenine, **(C)** 500 μM hypoxanthine, **(D)** 100 μM hypoxanthine, **(E)** 150 μM guanine, **(F)** 100 μM guanine, **(G)** 300 μM xanthine, **(H)** 300 μM xanthine, **(I)** 300 μM uric acid, **(J)** 300 μM uric acid, **(K)** 500 μM allantoin, or **(L)** 500 μM allantoin. The leftward pointing arrow above the expression strains demonstrates the expectation that expression of relevant transporters will improve growth on a compound as a sole nitrogen source. The rightward pointing arrow above the knockout strains demonstrates the expectation that knocking out relevant transporters will impair growth on a compound as a sole nitrogen source. NI indicates control wells in which bacteria were not inoculated. For each expression strain, 1 well per substrate per experiment from 3 independent experiments (n=3) was included. For each knockout strain, 1 well per substrate per experiment from 4 independent experiments (n=4) was included, except for adenine condition in which 1 well per experiment from 3 independent experiments (n=3) was included. Data represent mean ± SE. For statistical analysis, area under curve (AUC) values of all strains tested in an experiment were compared using a repeated measures one-way ANOVA with Dunnett’s multiple comparison test. Detailed statistical comparisons are available in the main text.


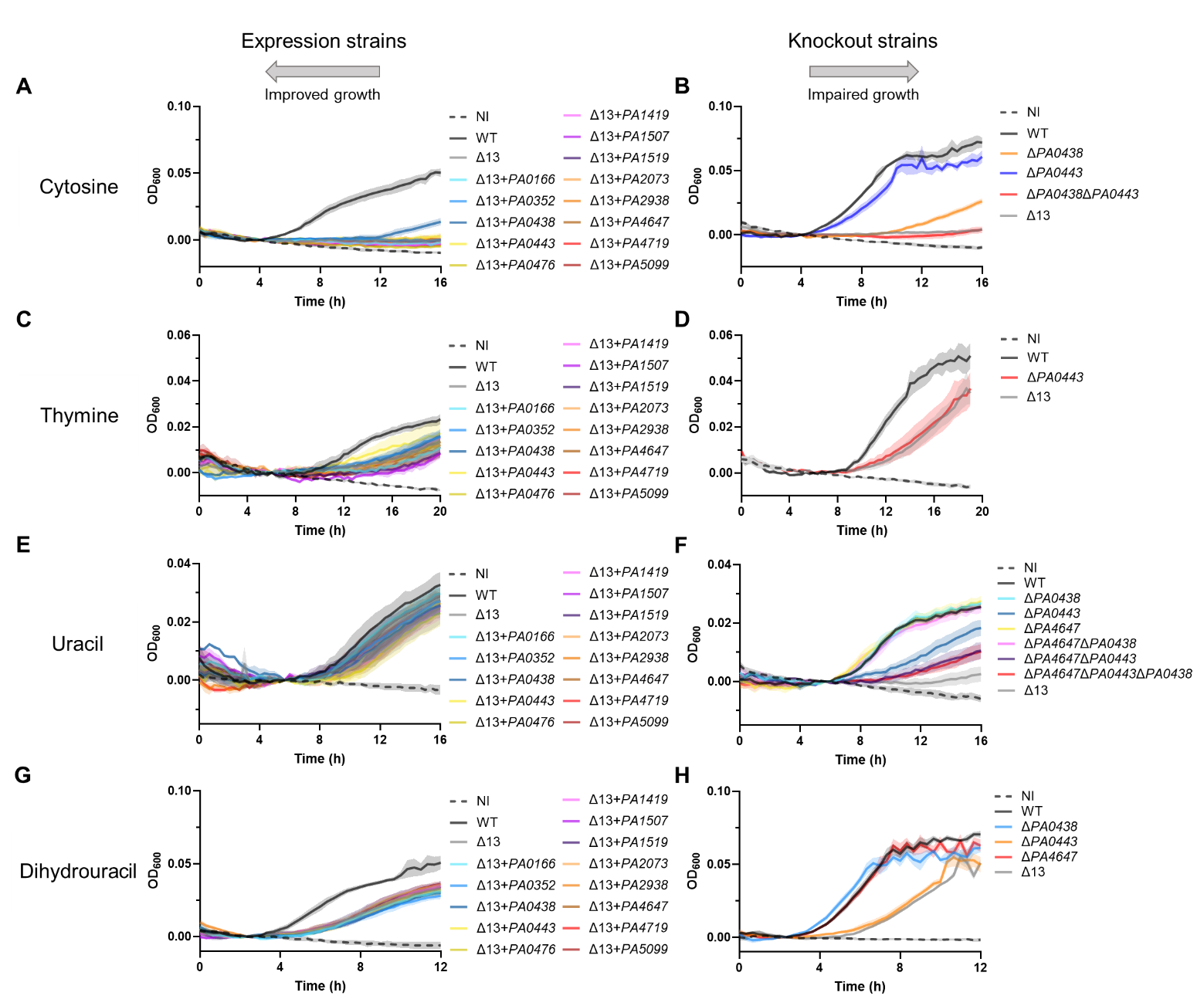


**Supplementary Figure 3. Growth of expression and knockout strains on pyrimidines as sole nitrogen sources enabled substrate identification of NCS1 and NCS2 transporters.** Growth of selected expression and knockout strains in nitrogen-free M9 supplemented with **(A)** 500 μM cytosine, **(B)** 500 μM cytosine, **(C)** 500 μM thymine, **(D)** 500 μM thymine, **(E)** 500 μM uracil, **(F)** 100 μM uracil, **(G)** 500 μM dihydrouracil, or **(H)** 500 μM dihydrouracil. The leftward pointing arrow above the expression strains demonstrates the expectation that expression of relevant transporters will improve growth on a compound as a sole nitrogen source. The rightward pointing arrow above the knockout strains demonstrates the expectation that knocking out relevant transporters will impair growth on a compound as a sole nitrogen source. NI indicates control wells in which bacteria were not inoculated. For each expression strain, 1 well per substrate per experiment from 3 independent experiments (n=3) was included. For each knockout strain, 1 well per substrate per experiment from 4 independent experiments (n=4) was included. Data represent mean ± SE. For statistical analysis, area under curve (AUC) values of all strains tested in an experiment were compared using a repeated measures one-way ANOVA with Dunnett’s multiple comparison test. Detailed statistical comparisons are available in the main text.


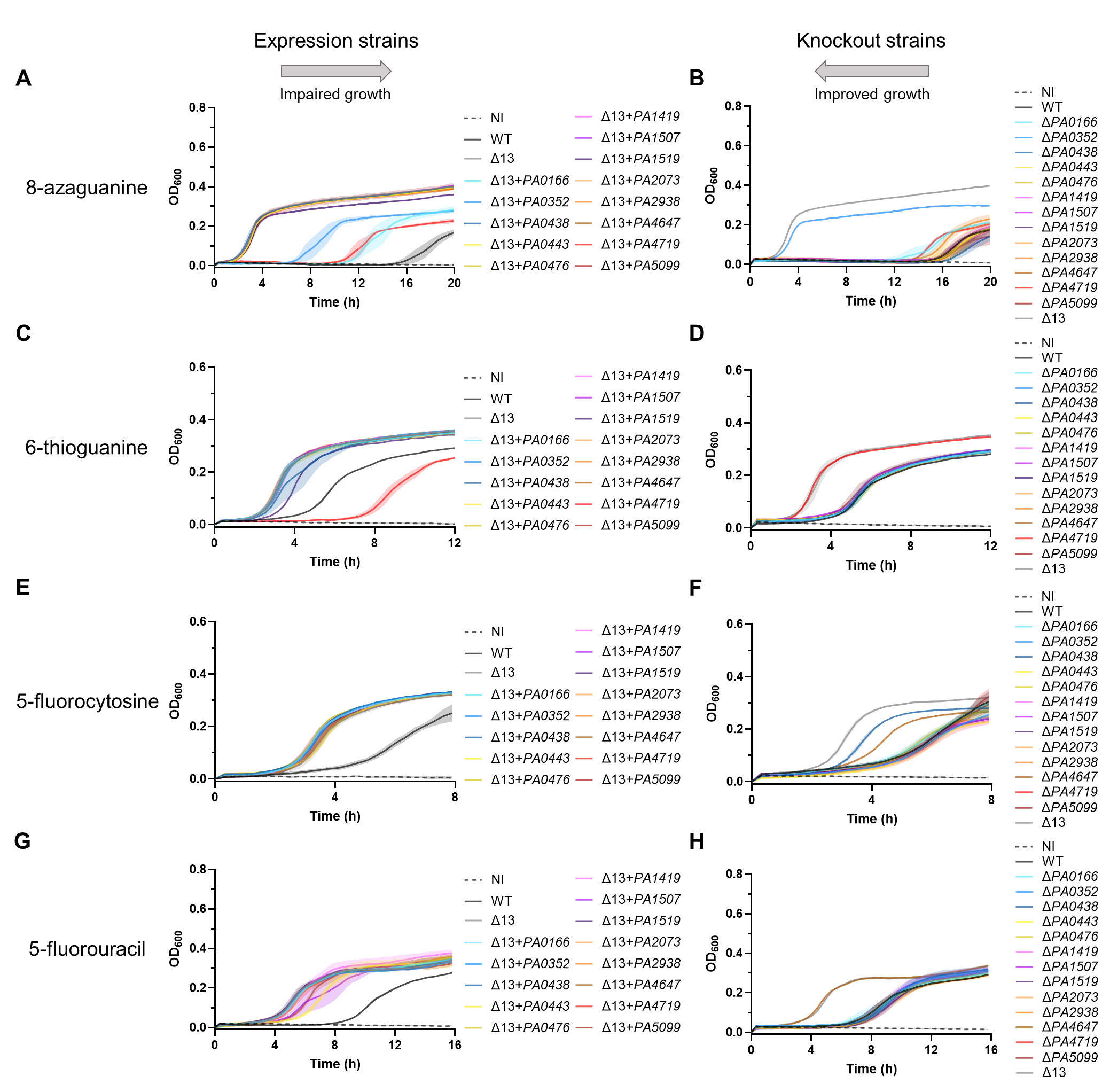


**Supplementary Figure 4. Growth of expression and knockout strains on nitrogen-replete media exposed to toxic nucleobase analogs enabled substrate identification of NCS1 and NCS2 transporters.** Growth of selected expression and knockout strains in M9 containing casamino acids and the toxic compounds **(A)** 1000 μM 8-azaguanine, **(B)** 1000 μM 8-azaguanine, **(C)** 100 μM 6-thioguanine, **(D)** 100 μM 6-thioguanine, **(E)** 1000 μM 5-fluorocytosine, **(F)** 1000 μM 5-fluorocytosine, **(G)** 10 μM 5-fluorouracil, **(H)** 10 μM 5-fluorouracil. The rightward pointing arrow above the expression strains demonstrates the expectation that expression of relevant transporters will impair growth when exposed to a toxic nucleobase analog. The leftward pointing arrow above the knockout strains demonstrates the expectation that knocking out relevant transporters will improve growth when exposed to a toxic nucleobase analog. NI indicates control wells in which bacteria were not inoculated. For each expression strain, 1 well per substrate per experiment from 3 independent experiments (n=3) was included unless otherwise stated due to removal of outliers. For 8-azaguanine expression experiments, n=2 for Δ13, PA2938, PA4647, and PA5099; for 6-thioguanine expression experiments, n=2 for PA0476, PA1419, and PA1507 and n=1 for PA1519 and PA2073; for 5-fluorocytosine expression experiments, n=2 for Δ13, PA0166, PA1519, PA2073, and PA4719; for 5-fluorouracil expression experiments, n=2 for PA0352, PA0438, PA0476, and PA1519. For each knockout strain, 1 well per substrate per experiment from 4 independent experiments (n=4) was included unless otherwise stated due to removal of outliers. For 8-azaguanine knockout experiments, n=3 for ΔPA0443; for 6-thioguanine knockout experiments, n=3 for WT, ΔPA0166, ΔPA0438, ΔPA0443, ΔPA1419, ΔPA1507, ΔPA4719 and n=2 for ΔPA0352, ΔPA5099, and Δ13; for 5-fluorocytosine knockout experiments, n=3 for ΔPA0438 and ΔPA0443 and n=2 for ΔPA4647 and Δ13; for 5-fluorouracil knockout experiments, n=2 for Δ13. Data represent mean ± SE. For statistical analysis, area under curve (AUC) values of all strains tested in an experiment were compared using a repeated measures one-way ANOVA with Dunnett’s multiple comparison test. Detailed statistical comparisons are available in the main text.


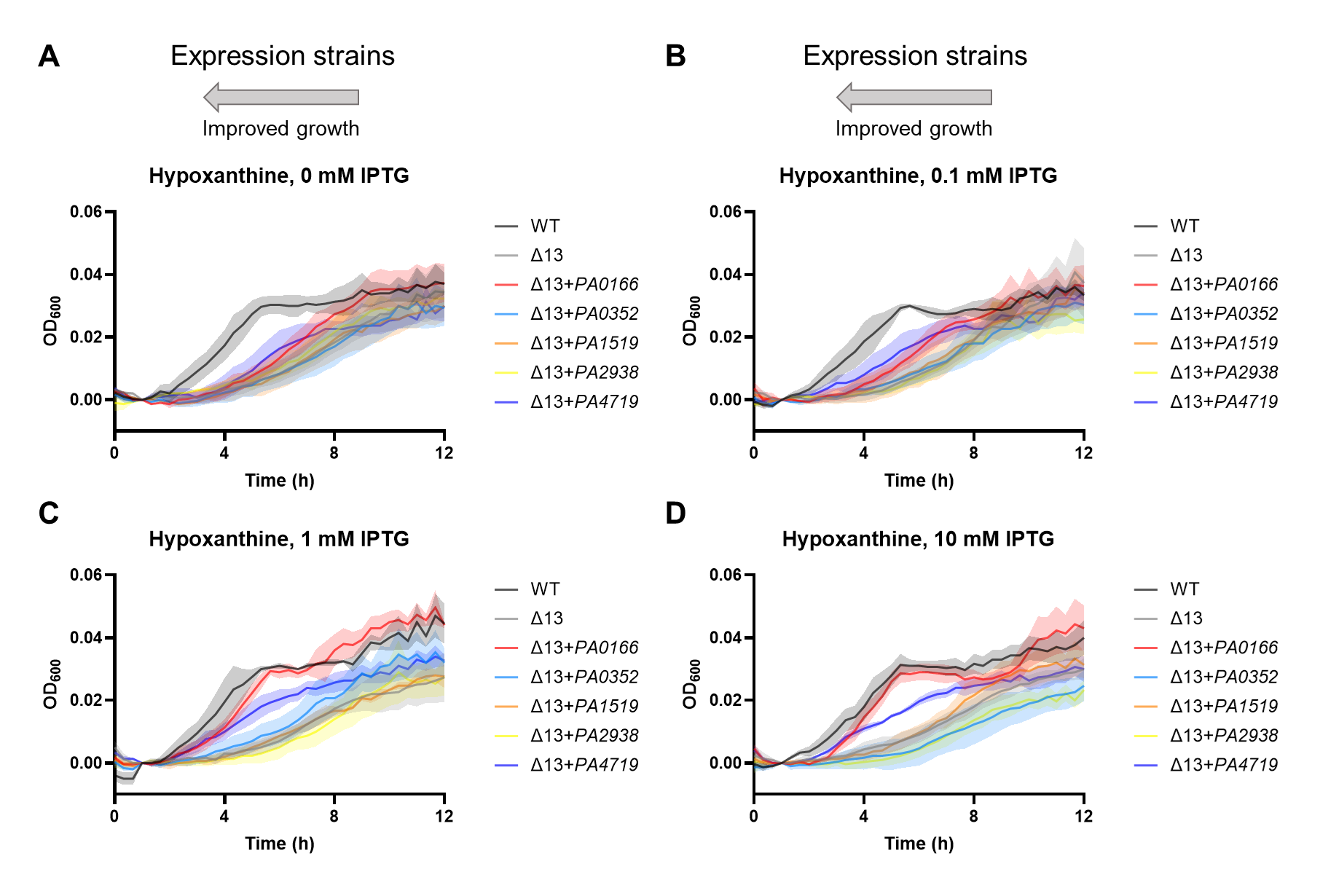


**Supplementary Figure 5. Growth of expression and knockout strains on hypoxanthine as a sole nitrogen source at different inducer concentrations.** Growth of selected expression strains in nitrogen-free M9 supplemented with 100 μM hypoxanthine and **(A)** 0 mM IPTG inducer **(B)** 0.1 mM IPTG inducer, **(C)** 1 mM IPTG inducer, **(D)** 10 mM IPTG inducer. The leftward pointing arrow above the expression strains demonstrates the expectation that expression of relevant transporters will improve growth on a compound as a sole nitrogen source. For each expression strain, 1 well per substrate per experiment from 3 independent experiments (n=3) was included unless otherwise stated due to removal of outliers. For 1 mM IPTG condition, n=2 for WT. Data represent mean ± SE. For statistical analysis, area under curve (AUC) values of all strains tested in an experiment were compared using a repeated measures one-way ANOVA with Dunnett’s multiple comparison test.


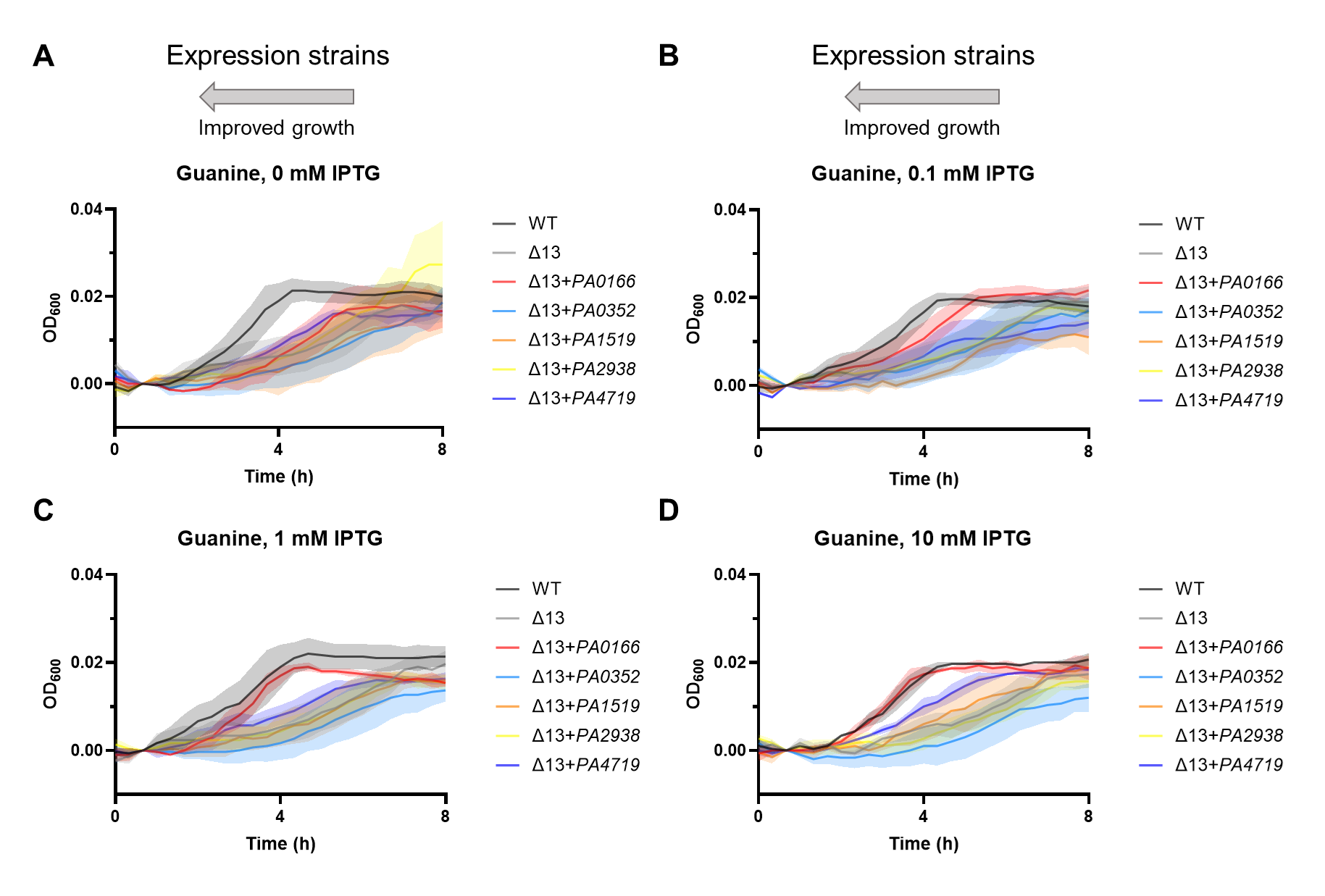


**Supplementary Figure 6. Growth of expression and knockout strains on guanine as a sole nitrogen source at different inducer concentrations.** Growth of selected expression strains in nitrogen-free M9 supplemented with 100 μM guanine and **(A)** 0 mM IPTG inducer **(B)** 0.1 mM IPTG inducer, **(C)** 1 mM IPTG inducer, **(D)** 10 mM IPTG inducer. The leftward pointing arrow above the expression strains demonstrates the expectation that expression of relevant transporters will improve growth on a compound as a sole nitrogen source. For each expression strain, 1 well per substrate per experiment from 3 independent experiments (n=3) was included unless otherwise stated due to removal of outliers. For 10 mM IPTG condition, n=2 for PA1519. Data represent mean ± SE. For statistical analysis, area under curve (AUC) values of all strains tested in an experiment were compared using a repeated measures one-way ANOVA with Dunnett’s multiple comparison test.


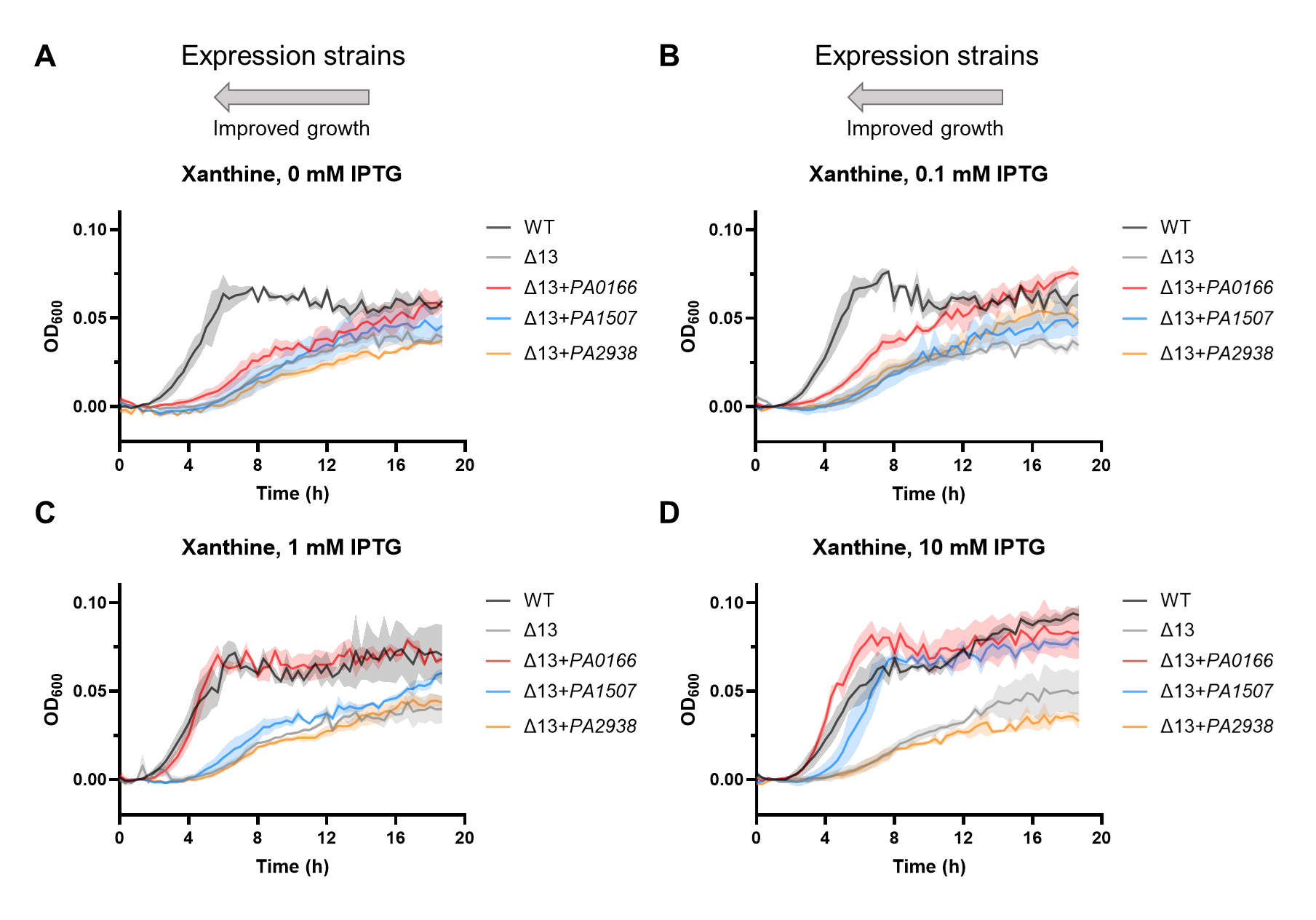


**Supplementary Figure 7. Growth of expression and knockout strains on xanthine as a sole nitrogen source at different inducer concentrations.** Growth of selected expression strains in nitrogen-free M9 supplemented with 300 μM xanthine and **(A)** 0 mM IPTG inducer **(B)** 0.1 mM IPTG inducer, **(C)** 1 mM IPTG inducer, **(D)** 10 mM IPTG inducer. The leftward pointing arrow above the expression strains demonstrates the expectation that expression of relevant transporters will improve growth on a compound as a sole nitrogen source. For each expression strain, 1 well per substrate per experiment from 3 independent experiments (n=3) was included. Data represent mean ± SE. For statistical analysis, area under curve (AUC) values of all strains tested in an experiment were compared using a repeated measures one-way ANOVA with Dunnett’s multiple comparison test.


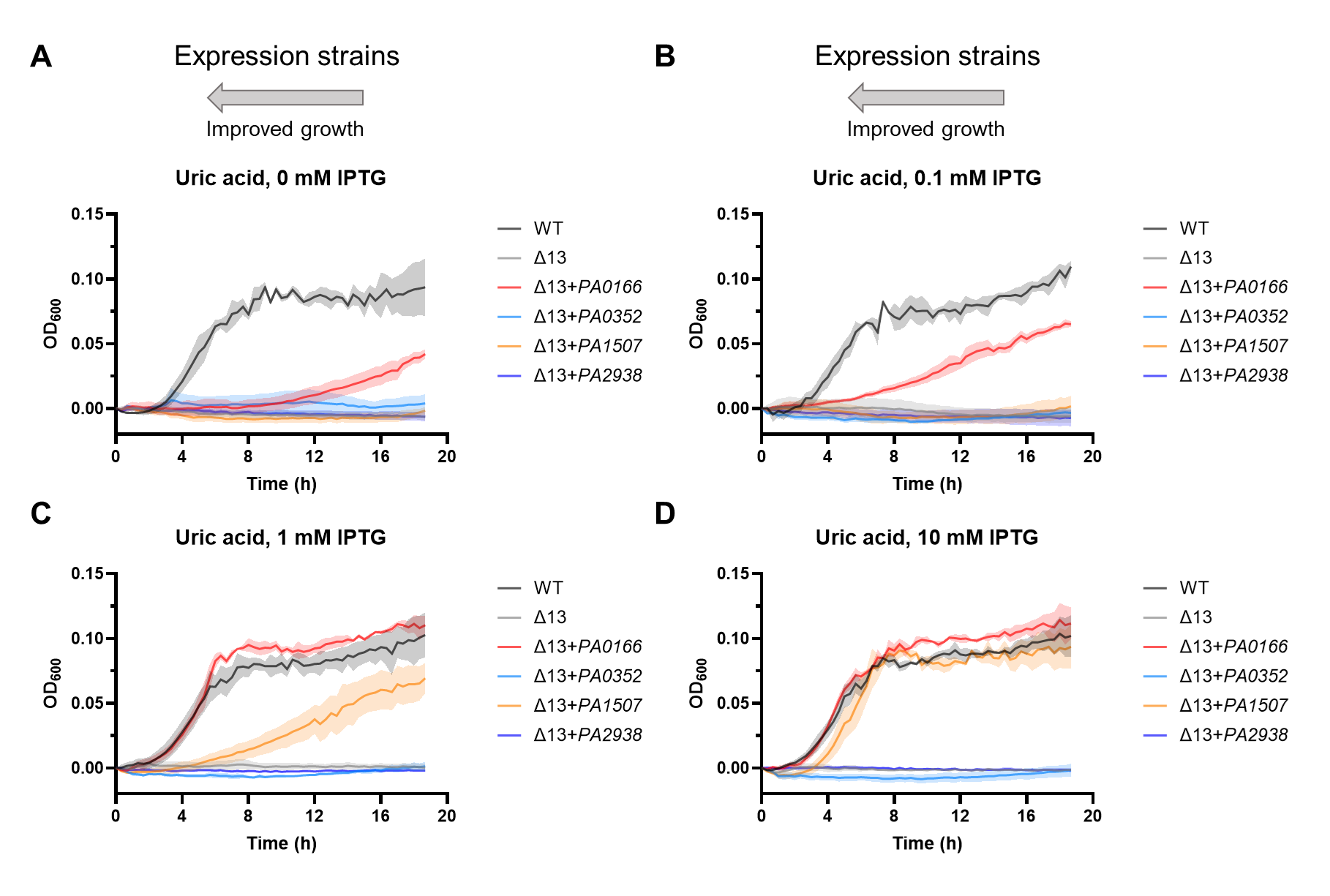


**Supplementary Figure 8. Growth of expression and knockout strains on uric acid as a sole nitrogen source at different inducer concentrations.** Growth of selected expression strains in nitrogen-free M9 supplemented with 300 μM uric acid and **(A)** 0 mM IPTG inducer **(B)** 0.1 mM IPTG inducer, **(C)** 1 mM IPTG inducer, **(D)** 10 mM IPTG inducer. The leftward pointing arrow above the expression strains demonstrates the expectation that expression of relevant transporters will improve growth on a compound as a sole nitrogen source. For each expression strain, 1 well per substrate per experiment from 3 independent experiments (n=3) was included. Data represent mean ± SE. For statistical analysis, area under curve (AUC) values of all strains tested in an experiment were compared using a repeated measures one-way ANOVA with Dunnett’s multiple comparison test.


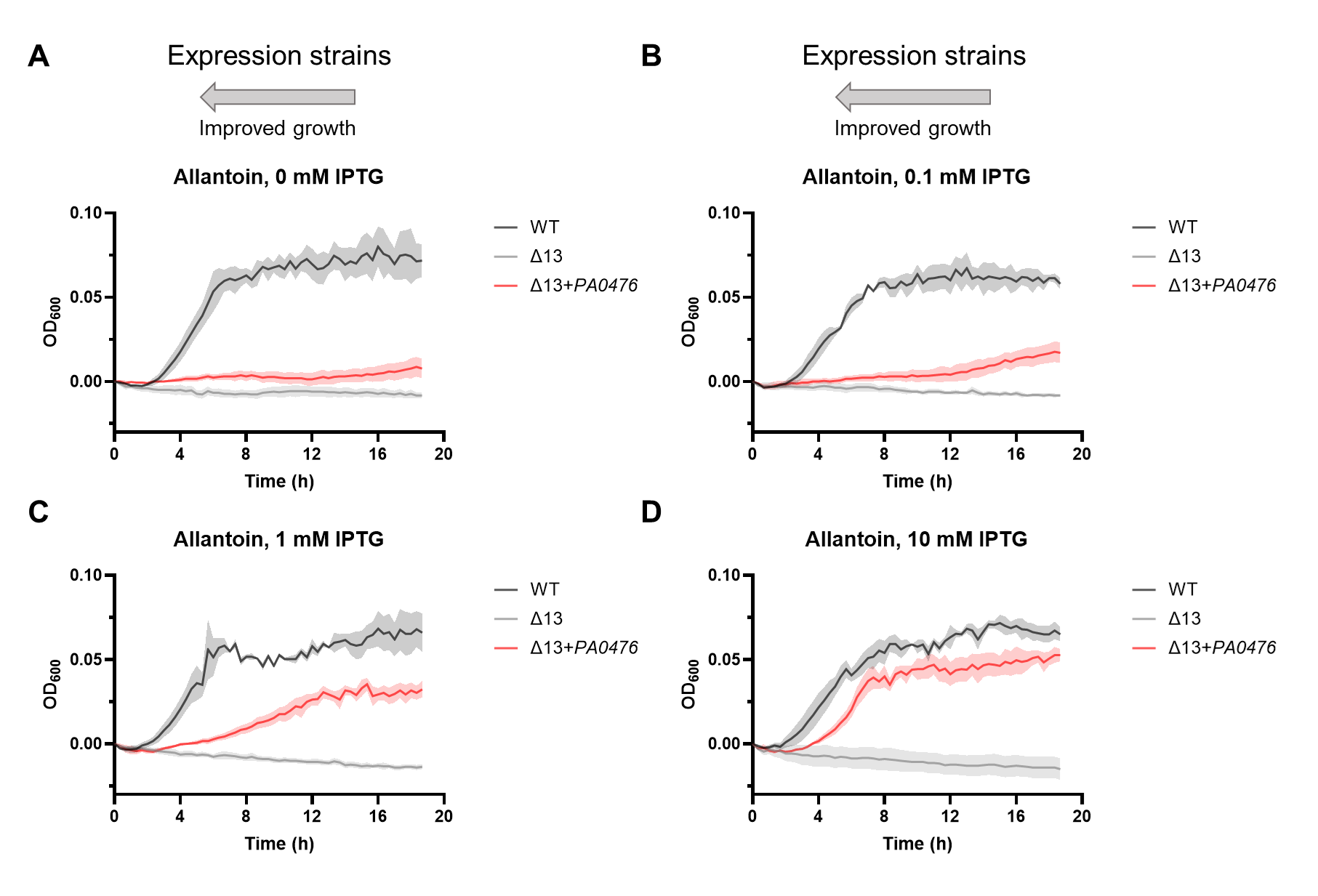


**Supplementary Figure 9. Growth of expression and knockout strains on allantoin as a sole nitrogen source at different inducer concentrations.** Growth of selected expression strains in nitrogen-free M9 supplemented with 500 μM allantoin and **(A)** 0 mM IPTG inducer **(B)** 0.1 mM IPTG inducer, **(C)** 1 mM IPTG inducer, **(D)** 10 mM IPTG inducer. The leftward pointing arrow above the expression strains demonstrates the expectation that expression of relevant transporters will improve growth on a compound as a sole nitrogen source. For each expression strain, 1 well per substrate per experiment from 3 independent experiments (n=3) was included. Data represent mean ± SE. For statistical analysis, area under curve (AUC) values of all strains tested in an experiment were compared using a repeated measures one-way ANOVA with Dunnett’s multiple comparison test.


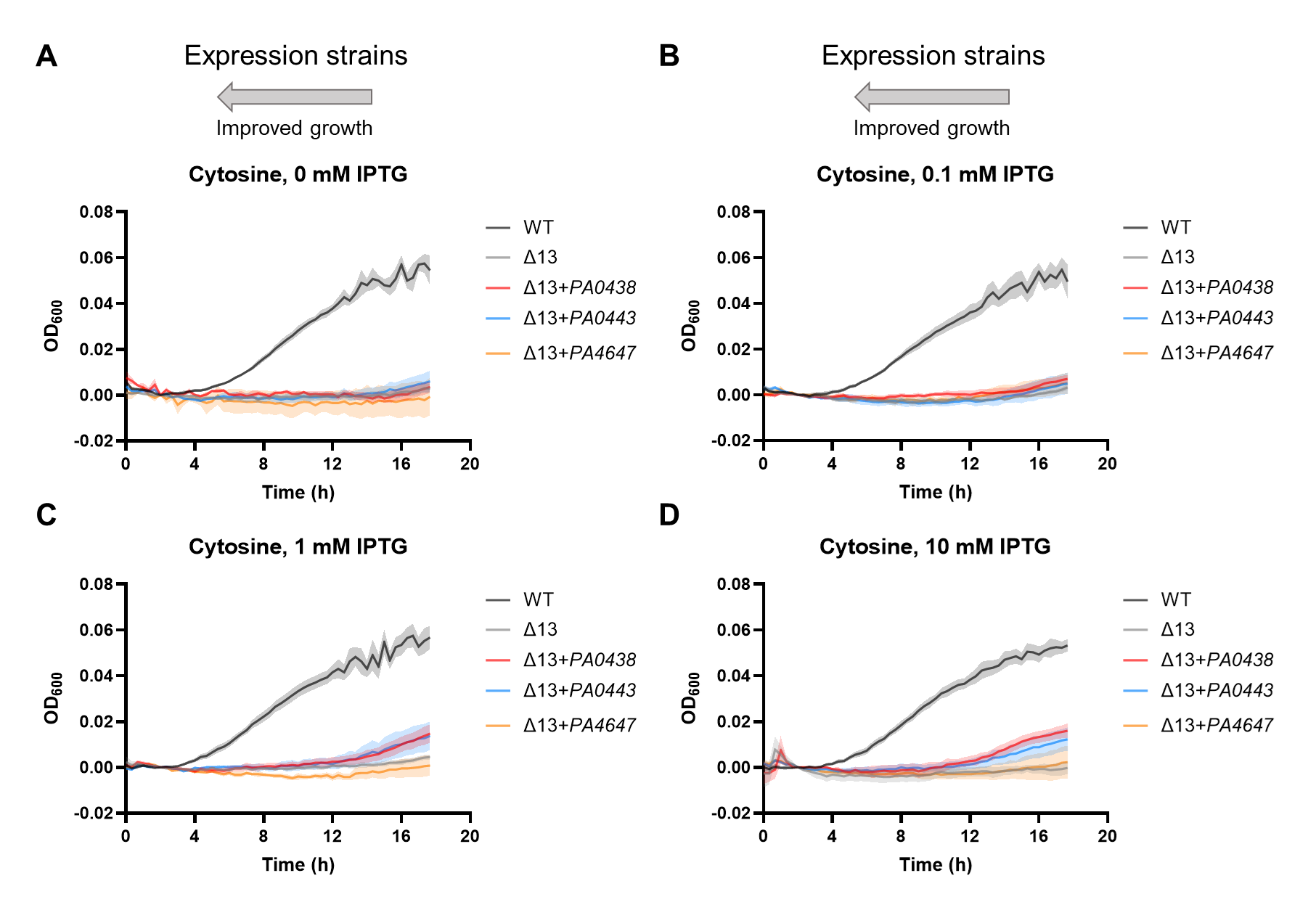


**Supplementary Figure 10. Growth of expression and knockout strains on cytosine as a sole nitrogen source at different inducer concentrations.** Growth of selected expression strains in nitrogen-free M9 supplemented with 500 μM cytosine and **(A)** 0 mM IPTG inducer **(B)** 0.1 mM IPTG inducer, **(C)** 1 mM IPTG inducer, **(D)** 10 mM IPTG inducer. The leftward pointing arrow above the expression strains demonstrates the expectation that expression of relevant transporters will improve growth on a compound as a sole nitrogen source. For each expression strain, 1 well per substrate per experiment from 4 independent experiments (n=4) was included. Data represent mean ± SE. For statistical analysis, area under curve (AUC) values of all strains tested in an experiment were compared using a repeated measures one-way ANOVA with Dunnett’s multiple comparison test.


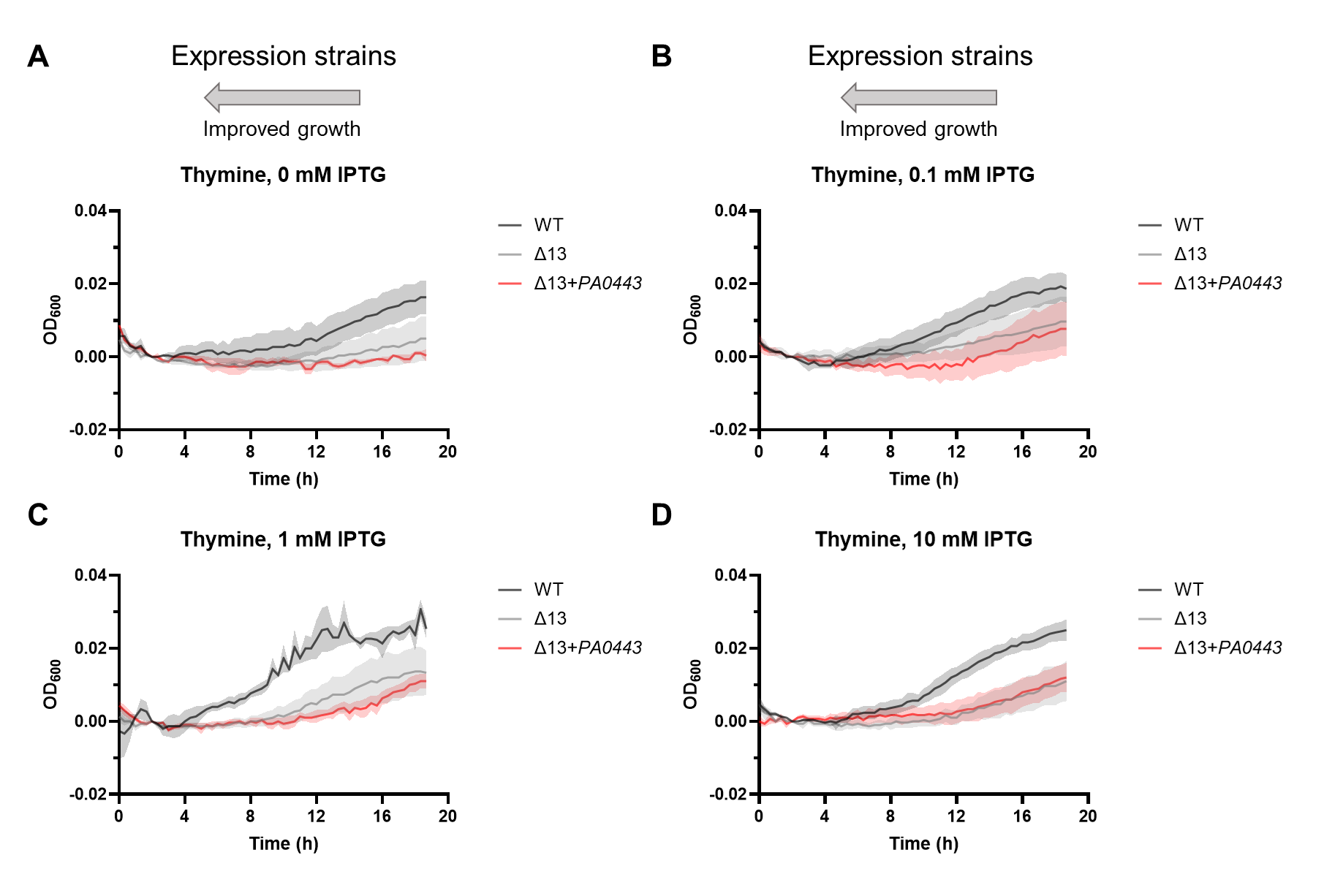


**Supplementary Figure 11. Growth of expression and knockout strains on thymine as a sole nitrogen source at different inducer concentrations.** Growth of selected expression strains in nitrogen-free M9 supplemented with 500 μM thymine and **(A)** 0 mM IPTG inducer **(B)** 0.1 mM IPTG inducer, **(C)** 1 mM IPTG inducer, **(D)** 10 mM IPTG inducer. For each expression strain, 1 well per substrate per experiment from 3 independent experiments (n=3) was included. Data represent mean ± SE. The leftward pointing arrow above the expression strains demonstrates the expectation that expression of relevant transporters will improve growth on a compound as a sole nitrogen source. For statistical analysis, area under curve (AUC) values of all strains tested in an experiment were compared using a repeated measures one-way ANOVA with Dunnett’s multiple comparison test.


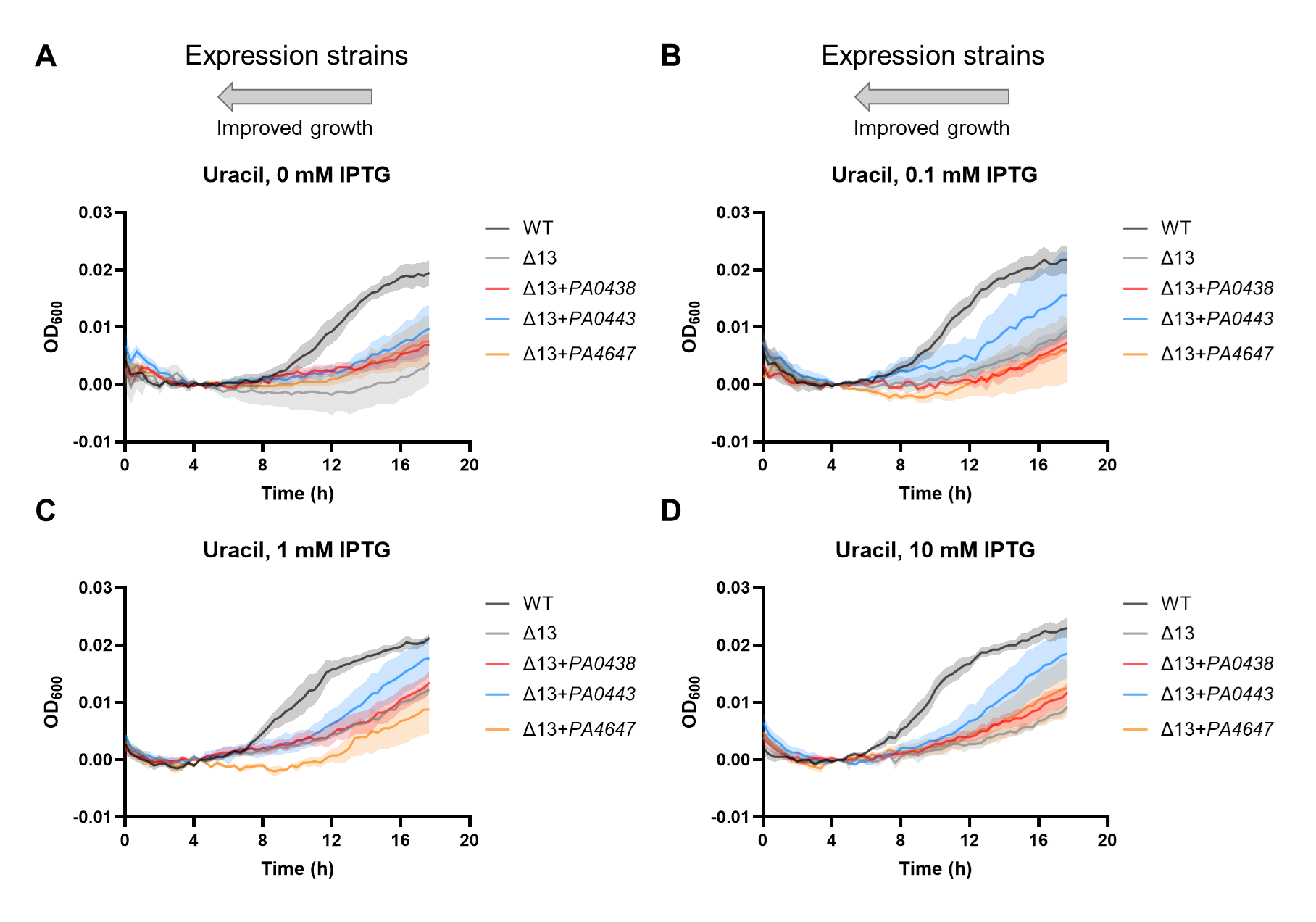


**Supplementary Figure 12. Growth of expression and knockout strains on uracil as a sole nitrogen source at different inducer concentrations.** Growth of selected expression strains in nitrogen-free M9 supplemented with 100 μM uracil and **(A)** 0 mM IPTG inducer **(B)** 0.1 mM IPTG inducer, **(C)** 1 mM IPTG inducer, **(D)** 10 mM IPTG inducer. The leftward pointing arrow above the expression strains demonstrates the expectation that expression of relevant transporters will improve growth on a compound as a sole nitrogen source. For each expression strain, 1 well per substrate per experiment from 4 independent experiments (n=4) was included. Data represent mean ± SE. For statistical analysis, area under curve (AUC) values of all strains tested in an experiment were compared using a repeated measures one-way ANOVA with Dunnett’s multiple comparison test.


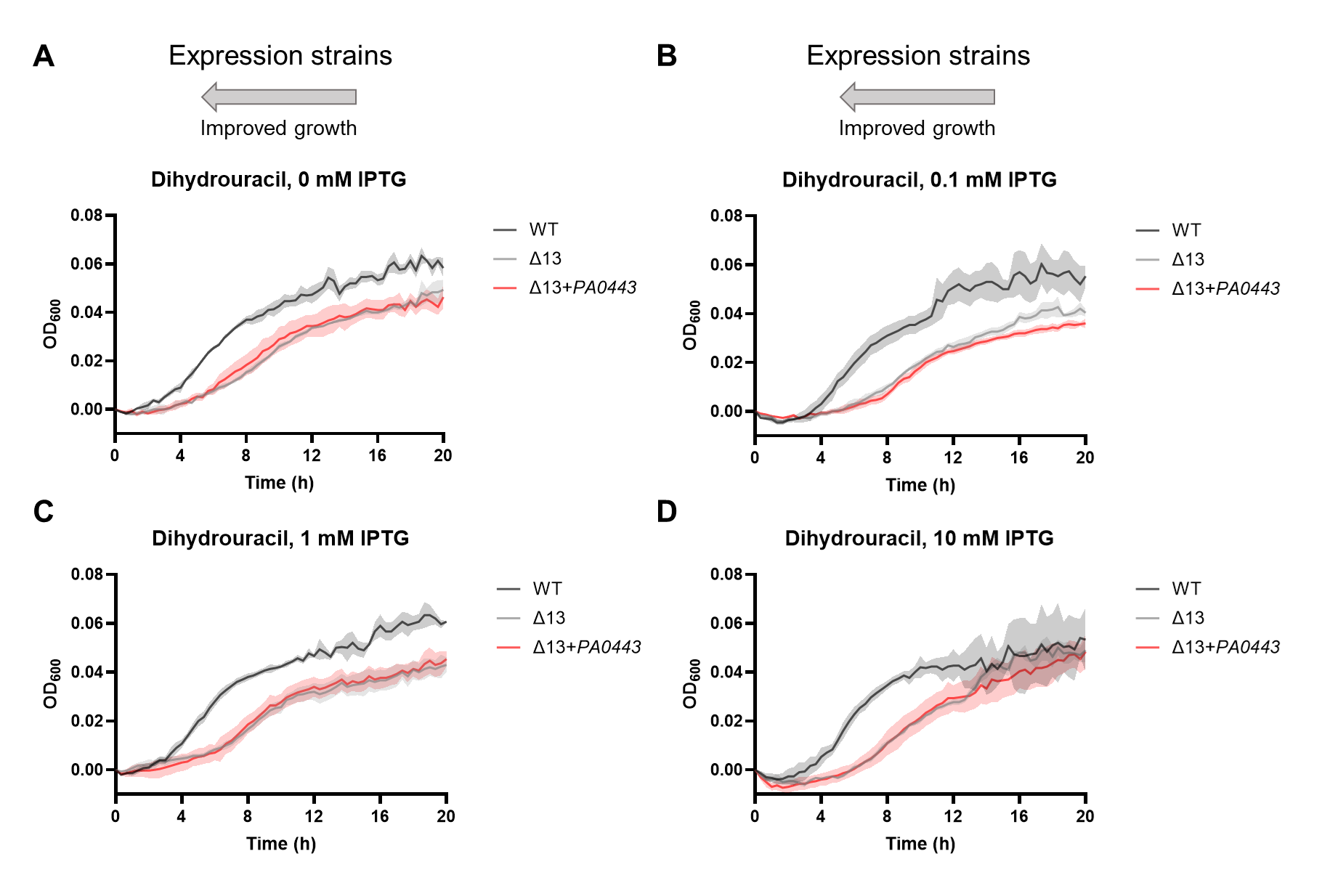


**Supplementary Figure 13. Growth of expression and knockout strains on dihydrouracil as a sole nitrogen source at different inducer concentrations.** Growth of selected expression strains in nitrogen-free M9 supplemented with 500 μM dihydrouracil and **(A)** 0 mM IPTG inducer **(B)** 0.1 mM IPTG inducer, **(C)** 1 mM IPTG inducer, **(D)** 10 mM IPTG inducer. The leftward pointing arrow above the expression strains demonstrates the expectation that expression of relevant transporters will improve growth on a compound as a sole nitrogen source. For each expression strain, 1 well per substrate per experiment from 3 independent experiments (n=3) was included. Data represent mean ± SE. For statistical analysis, area under curve (AUC) values of all strains tested in an experiment were compared using a repeated measures one-way ANOVA with Dunnett’s multiple comparison test.
